# DyNAMiC Workbench: an integrated development environment for dynamic DNA nanotechnology

**DOI:** 10.1098/rsif.2015.0580

**Published:** 2015-10-06

**Authors:** Casey Grun, Justin Werfel, David Yu Zhang, Peng Yin

**Affiliations:** 1 Wyss Institute for Biologically Inspired Engineering, Harvard University, Boston, MA, USA; 2 Department of Systems Biology, Harvard Medical School, Boston, MA, USA

**Keywords:** DNA nanotechnology, molecular programming, self-assembly, software, sequence design, integrated development environment

## Abstract

Dynamic DNA nanotechnology provides a promising avenue for implementing sophisticated assembly processes, mechanical behaviours, sensing and computation at the nanoscale. However, design of these systems is complex and error-prone, because the need to control the kinetic pathway of a system greatly increases the number of design constraints and possible failure modes for the system. Previous tools have automated some parts of the design workflow, but an integrated solution is lacking. Here, we present software implementing a three ‘tier’ design process: a high-level visual programming language is used to describe systems, a molecular compiler builds a DNA implementation and nucleotide sequences are generated and optimized. Additionally, our software includes tools for analysing and ‘debugging’ the designs *in silico*, and for importing/exporting designs to other commonly used software systems. The software we present is built on many existing pieces of software, but is integrated into a single package—accessible using a Web-based interface at http://molecular-systems.net/workbench. We hope that the deep integration between tools and the flexibility of this design process will lead to better experimental results, fewer experimental design iterations and the development of more complex DNA nanosystems.

## 1. Introduction

DNA has been demonstrated to be a robust and versatile substrate for engineering static nanostructures [1,2] and dynamic nanodevices [3,4]. The specificity of Watson–Crick base pairing [5,6], combined with recent improvements in thermodynamic predictive models [7,8] and rapidly decreasing costs for commercial oligonucleotide synthesis [9], have resulted in an explosion of research in DNA nanotechnology [10], in which hybridization interactions (rather than enzymatic processes) are primarily used to implement the desired molecular behaviour. Recently, the field has progressed beyond primarily demonstrations of static equilibrium structure formation to the design of dynamic systems with kinetically controlled, non-equilibrium dynamics [4]—including molecular machines [11,12], motors [13,14], walkers [15–17], amplifiers [16,18,19], self-assembly processes [16,20], logic circuits [21,22] and other sophisticated computational devices [23,24].

Design of dynamic nucleic acid systems—both at the high level of abstraction and at the low level of sequence design—requires consideration of a different set of design parameters and metrics from the design of static DNA nanostructures. Static structures are designed to adopt a single, minimum free energy (MFE) structure; dynamic systems additionally require design of a kinetic pathway—a series of reactions. Disruption of any of these intended reactions or prevalence of unintended ‘side’ reactions can disturb the intended behaviour. For instance, poor kinetics of individual reactions can lead to slow performance for a molecular calculation; unanticipated ‘leak’ reactions can cause unexpected product formation; and side reactions that produce undesired products can result in low yields of the intended product. These problems are amplified in more complex systems, since the number of possible unintended interactions grows at least quadratically with the number of species. Recent demonstrations of nucleic acid logic circuits now exceed over 100 different molecular species [23,24], motivating the need for novel software to assist the design of complex systems.

**Figure 1.**
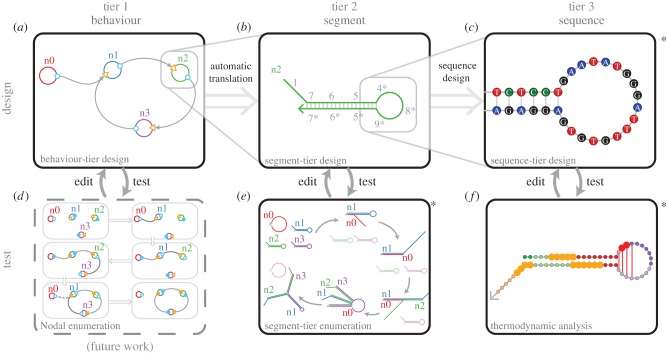
Tiers of abstraction and the overview of the design workflow. The design process occurs at three ‘tiers’ of decreasing abstraction: at the level of (1) behavioural description, (2) segment-level complementarity design and (3) nucleotide sequences. The design workflow involves two forms of tools: (*a*–*c*) describe tools for *creating* dynamic nucleic acid systems, while panels (*d*–*f*) describe tools for *analysing and testing* these systems *in silico*. Box (*d*) is future work, not yet incorporated by DyNAMiC Workbench; asterisks denote tools that use previously described software. Images in all panels except (*d*,*f*) were generated automatically by DyNAMiC Workbench. (*a*) Users begin by describing an abstract behaviour, using a formalism such as the Nodal language of Yin *et al*. [16] (future extensions may incorporate alternative behavioural designers). A built-in compiler automatically generates a segment-level representation (when possible) of a system of nucleic acid strands which implements the desired behaviour, through a systematic labelling of segments corresponding to the nodes and connections specified (see §3.1). (*b*) A segment-tier design (specifying the identity and complementarity relationships between all segments in the system) can be either generated from the behavioural designer or assembled directly by the user (see §3.2). (*c*) From a segment-level design, nucleotide sequences can be generated with one of a variety of sequence design packages. DyNAMiC Workbench allows users to interact with and modify systems at all three levels (see §3.3). (*d*,*e*) Reaction enumerators identify all possible reaction paths, highlighting possible undesired interactions. Enumeration, in principle, can be performed at the behavioural tier among behavioural species, or at the segment tier among molecular species. The results of both enumerations should be comparable, but the segment tier enumeration may reveal unintended side-reactions or kinetic traps not prescribed by the behavioural enumeration (see §3.4.1). (*f*) Base-tier sequences can be analysed using thermodynamic methods to identify unintended secondary structure in monomers [25], as well as some unintended interactions between species (see §3.4.2).

To describe the design process for a dynamic DNA system, we propose a system of three different ‘tiers’ of abstraction (figure 1). The first tier describes desired system behaviour, using a set of high-level abstractions that represent molecular species and their interactions (figure 1*a*). The second tier gives a set of idealized DNA strands and interactions (in terms of prescribed regions of Watson–Crick complementarities) that implement the first-tier behavioural species (figure 1*b*). The lowest tier gives specific sequences of nucleotides to implement the prescribed complementarities among the second-tier species (figure 1*c*). In principle, one may begin the design process at any of the tiers and software should automatically translate to lower tiers; similarly, computer-aided verification at any tier should be possible. In practice, the first two tiers are traditionally designed by hand, and the final ‘sequence design’ step uses one of a variety of computational sequence optimization [26–30] and verification [25,31–33] packages; that is, only sequence-tier design and analysis is automated.

Recent software packages have made impressive advances in automating and integrating various parts of this design process. Visual DSD [34,35] has been used to automate the ‘segment-level’ (tier 2) design and analysis of systems containing hundreds of distinct species [25,34]. However, Visual DSD currently does not support the design and enumeration of branched junction structures (used in many demonstrations of self-assembly [16] and molecular computation [21]), nor does it support enumeration of certain reaction types, such as four-way branch migration or branch migration with remote toeholds [36,37]. Qian & Winfree [22,23] have presented a compiler for their ‘seesaw gate’ systems, which has been used in demonstrations of sophisticated molecular computation; however, this abstraction is built around a single structural motif (the seesaw gate) and is therefore of limited use for applications in self-assembly that require a wider variety of structural features (e.g. hairpins, branched junctions, etc.). The NUPACK software package [8,25,30] integrates thermodynamic design and evaluation of nucleotide sequences; however, it does not currently provide a programming language for tier 1 (behavioural) design, nor does it allow for analysis or evaluation at the segment tier. Therefore, no current package integrates a full-featured DNA programming language with sequence designers, as well as analysis and verification tools at the segment and sequence tiers. See table 1 for a detailed comparison.

**Table 1.** Comparison of currently available software packages for dynamic DNA nanotechnology. ✓, feature is implemented by this package; ×, feature is not implemented by this package; and ✓*, feature is provided by an external package with which this package directly interfaces.

		DyNAMiC Workbench^a^	DSD [34]^a^	Seesaw Compiler [22]^a^	NUPACK [25]^a^	Mfold [38]^a^	Vienna RNAfold [32]^a^	Multistrand [39]^a^
implementation	behavioural	✓	✓	✓	×	×	×	×
	segment	✓	✓	✓	✓	×	×	×
	sequence	✓*	✓	✓	✓	×	✓	×
reaction enumeration	behavioural	×	×	×	×	×	×	×
	segment	✓*	✓	×	×	×	×	×
	sequence	×	×	×	×	×	×	✓
kinetic simulation	behavioural	×	×	×	×	×	×	×
	segment	×	✓	✓*	×	×	×	×
	sequence	×	×	×	×	×	×	✓
thermodynamic simulation	structure prediction	✓*	×	×	✓	✓	✓	×
	partition function	✓*	×	×	✓	✓	✓	×

^a^Package is available as an online Web service at the time of publication.

Here, we present DyNAMiC Workbench (the **Dy**namic **N**ucleic **A**cid **M**echan**i**sm **C**ompiler), which provides a tightly integrated graphical user interface for all three tiers of design, as well as automated enumeration and analysis of potential interactions at the segment and sequence tiers. Users may begin by designing an abstract behaviour for a dynamic system and are assisted in translating that behaviour first to segments, then to nucleotide sequences. In this way, the design process is *hierarchical*, progressing through several layers of abstraction towards the ultimate implementation. However, the user may easily enter or exit the software at any stage of the design process, perform analysis *in silico* and make changes to the design based on the results of those tests. In this way, the design process can be *iterative*.

Furthermore, DyNAMiC Workbench supports integration with a number of commonly used software packages for each tier, allowing the user to ‘mix-and-match’ different aspects of preferred software. Finally, the system also includes a pluggable framework for expansion and inclusion of new tools and interfaces—for instance, a kinetic simulation package could be easily added. The software is deployed as a free Web service, available at http://molecular-systems.net/workbench, providing a cross-platform graphical interface without requiring installation of new software; a downloadable version is also available (at the same URL) to support local installations if preferred. We believe that the inherent flexibility in this design process, combined with the deep integration between the various tools, will help eventually enable fully automated design of dynamic systems. Expanded and improved *in silico* design and analysis will allow better experimental results with fewer experimental design iterations.

## 2. Designing a system with DyNAMiC Workbench

To illustrate the various features of DyNAMiC Workbench and the tiers of a typical design process for a dynamic nucleic acid system, we first present a sketch of the process for designing a triggered, catalytic three-arm branched junction, which has been experimentally demonstrated in [16]. We also provide four additional design examples in the electronic supplementary material.

**Figure 2.**
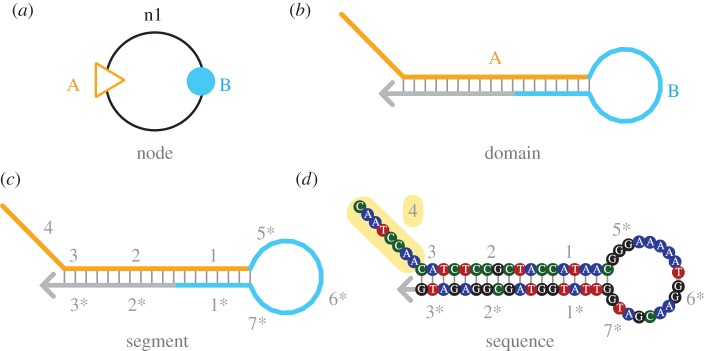
Abstractions and their definitions. (*a*) A strand or set of strands with a defined behaviour may be represented as a ‘node’, as described by Yin *et al*. [16]. In a node, domains are drawn as coloured ‘ports’. A triangle represents an ‘input port’, which can trigger opening of a circular ‘output port’. Output ports may in turn bind to downstream input ports. (*b*) Nucleic acid strands may be drawn as lines; behaviourally relevant portions of the molecule are called ‘domains’ and represented by capital letters; we have highlighted an input domain A in orange and an output domain B in blue—corresponding to the ports on the node pictured in panel (*a*). (*c*) ‘Segments’ represent contiguous regions of several nucleotides that act as discrete units of complementarity and are labelled by numbers or lowercase letters. For instance, segment 4 (highlighted in yellow in panel (*d*)) has the sequence ‘CAATCCAA’; each domain can comprise multiple segments. (*d*) Bubbles represent individual nucleotides; hashes indicate base pairing, and an arrow indicates the 3′ end of a strand.

**Figure 3.**
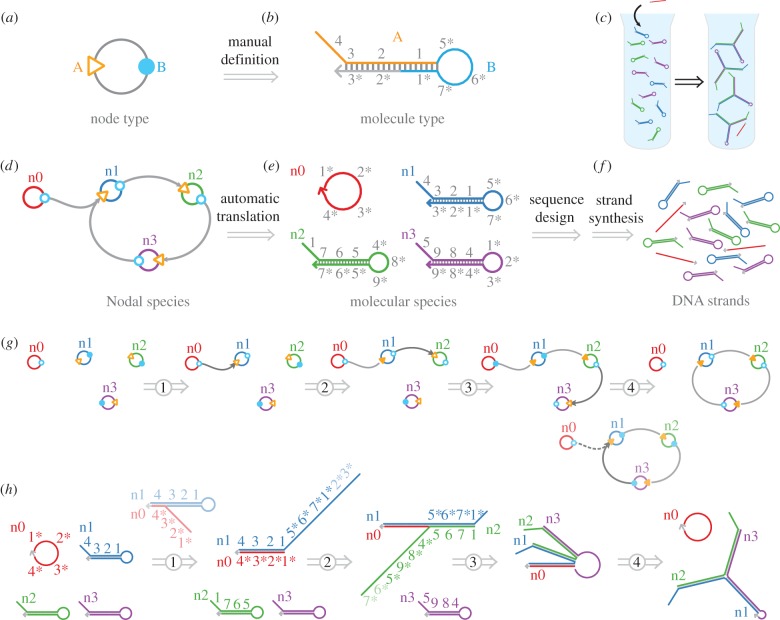
Design example. The Nodal formalism [16] maps (*a*) simple behavioural units (nodes) to (*b*) molecular architectures. Commonly used molecular motifs, or ‘molecule types’, may be expressed as node types by describing the primary and secondary structure of the molecule type and then assigning functional roles (e.g. input or output) to the molecule's domains. (*c*) Our target process is the conversion of a set of metastable hairpins into a three-arm junction, with a single-stranded initiator serving as a trigger. (*d*) Behavioural description of our catalytic three-arm branched junction, described by composing behavioural nodes. Once a node type has been defined, multiple Nodal species with the given node type may be instantiated and connected together. Each species of a given type will have the same basic structure, but will correspond to a distinct molecular species with a unique sequence identity. Connections between Nodal species (arrows) represent desired behavioural interactions between their domains. (*e*) These behavioural interactions also thus imply Watson–Crick complementarity relationships between the domains. DyNAMiC Workbench can use the node type definition to automatically map a set of Nodal species to a set of molecular species whose sequence complementarity relationships implement the intended behavioural interactions. This panel shows the segment-level representation of the hairpin monomer and initiator species which will make up our system. (*f*) Sequences may be designed to implement the molecular species, and DNA molecules may be produced by commercial oligonucleotide synthesis. (*g*) Intended ‘execution’ of the Nodal complementarity graph. (*h*) Segment-level enumeration of possible reactions between four starting complexes. Short, single-stranded regions at the hairpin termini serve as nucleation sites—‘toeholds’ to prime branch migration reactions. The opening of the hairpin by branch migration exposes new toeholds, implementing the cascade.

We adapt the ‘Nodal’ formalism [16] to describe the design procedure, following the three-tiered process outlined above. The terminology and abstractions of the Nodal formalism are summarized in figure 2. This formalism maps abstract behavioural units (‘node types’, figure 3*a*) to concrete molecular implementations (‘molecule types’, figure 3*b*)—DNA strands or complexes of strands. Once a node type has been defined, many ‘Nodal species’—instances of the node type—may be created. The process that we wish to demonstrate—triggered catalytic three-arm junction formation—is shown in figure 3*c*; we will implement this process by instantiating node types and composing Nodal species (figure 3*d*). The Nodal language allows a complicated behavioural process to be described by composing small, reusable modules, much like functions in a programming language (tier 1). DyNAMiC Workbench can then use the underlying node type definitions to automatically generate a complete segment-level (tier 2) molecular implementation (figure 3*e*). Ultimately, sequences are designed for each of these molecular species (tier 3), and many DNA molecules are produced for each species by commercial oligonucleotide synthesis (figure 3*f*).

The connectivity of our target structure (the three-arm junction)—along with our prescription that the assembly process be *stepwise, triggered* and *catalytic* (see below)—suggests a particular architecture of nodes. DyNAMiC Workbench currently provides a pool of approximately 20 pre-defined node types, based on previous work [16,20,40] (electronic supplementary material, figure S4); many of these node types have tested DNA implementations. For this example, we choose hairpins for the monomers and a linear strand without secondary structure for the initiator (node types m0 and m1). ‘Ports’ on the nodes represent ‘domains’ of the underlying molecular implementation (see figure 2 for terminology); the pattern of desired interactions between domains of the molecular species is encoded in the connections between ports on the nodes. Each domain in turn is subdivided into several ‘segments’—continuous regions of bases that are designed to act as discrete units. Figure 3*g* shows the intended execution of the Nodal reaction graph presented in figure 3*d*.

The target structure in this example, a three-arm junction (figure 3*c*), is designed to be formed in a kinetically guided pathway out of three monomer species and an initiator species (figure 3*e*). In the absence of the initiator, the monomers are to be metastable; this is indicated by drawing their output ports in a closed state (filled circles). Since the monomers are metastable, the initiator is required for target structure formation (‘triggered’). The Nodal language allows for two types of reactions: ‘assembly’ reactions occur between an open input and an open output port, while ‘disassembly’ reactions occur between an open output and a closed input. In the intended reaction pathway (figure 3*g*), the initiator n0 reacts with node n1 via an assembly reaction; this binding causes the output port on n1 (circle) to be switched from closed to open, reconfiguring n1 and allowing it to react with node n2 via another assembly reaction. The sequential opening of ports allows the assembly process for a single species of the target to proceed via several steps in a prescribed order (‘stepwise’). Once reacted with n2, a reconfigured n3 can displace the initiator n0 through a disassembly reaction; this reaction frees the initiator to react with another copy of node n1, implementing multiple turnovers (‘catalytic’).

This Nodal reaction pathway can be compiled to the more detailed reaction mechanism—shown at the segment level in figure 3*h* (compare each step to the Nodal mechanism in figure 3*g*). The initiator n0 first binds to hairpin n1 via the interaction between the ‘toehold’ segments 4 and 4* (where x* is the Watson–Crick complement of segment *x*). After n1 and n0 are thus co-localized, the 3, 2 and 1 segments on n0 can displace their analogues—hybridizing to the 3*, 2* and 1* segments—via a process known as ‘toehold-mediated branch migration’ [41–44]. At the conclusion of branch migration, monomer n1 is reconfigured so that the toehold segment 1* is no longer sequestered in the duplex and thus becomes available for a similar downstream reaction with hairpin n2. This process continues similarly for the remaining species.

The Nodal design can be easily constructed by the user via a drag-and-drop graphical interface. After completing the system design using the tier 1 Nodal formalism, the user invokes the DyNAMiC Workbench compiler, which automatically generates a valid set of tier 2 segment labels in order to implement the behaviour. The software can also identify potential interactions between molecules due to complementary segment interactions (‘reaction enumeration’)—including spurious reactions that may necessitate a redesign (§3.4.1). The reaction enumerator in DyNAMiC Workbench was used to generate the mechanism shown in figure 3*h*. The segment-tier (tier 2) descriptions capture all relevant structures and interactions of the system, assuming that all non-complementary segments have effectively orthogonal sequences (so as to not significantly interact with one another).

In reality, some degree of spurious interaction between segments is unavoidable, since real sequences are not perfectly orthogonal; careful sequence design is needed in order for the system to implement the behaviour as intended—both ensuring desired interactions are favourable and minimizing undesired side reactions that may occur between segments—both within or across different molecules [27]. For this step, DyNAMiC Workbench converts the segments generated in the previous tier into poly-N sequences of the proper lengths. The poly-N sequences are then mutated into a set of non-interacting sequences (a tier 3 design) using one of several existing software tools: Domain Design (DD) [29], NUPACK [25,30] or Multisubjective [45] (§3.3).

Finally, DyNAMiC Workbench interfaces with tools [25,32,38] to perform thermodynamic calculations as heuristic tests to assess the quality of the final sequences. For instance, we may compute the MFE structures of each of the strands or complexes in our ensemble to verify that they adopt the intended secondary structures in their monomeric form. Additionally, DyNAMiC Workbench can be instructed to look for strong pairwise interactions between species which are intended to be non-interacting. Once the user is satisfied that the sequences will implement the intended complementarity scheme, DyNAMiC Workbench can export the sequences of all relevant DNA strands; these sequences can then be directly provided to a commercial supplier of oligonucleotides for synthesis.

## 3. Methods and implementation

**Figure 4.**
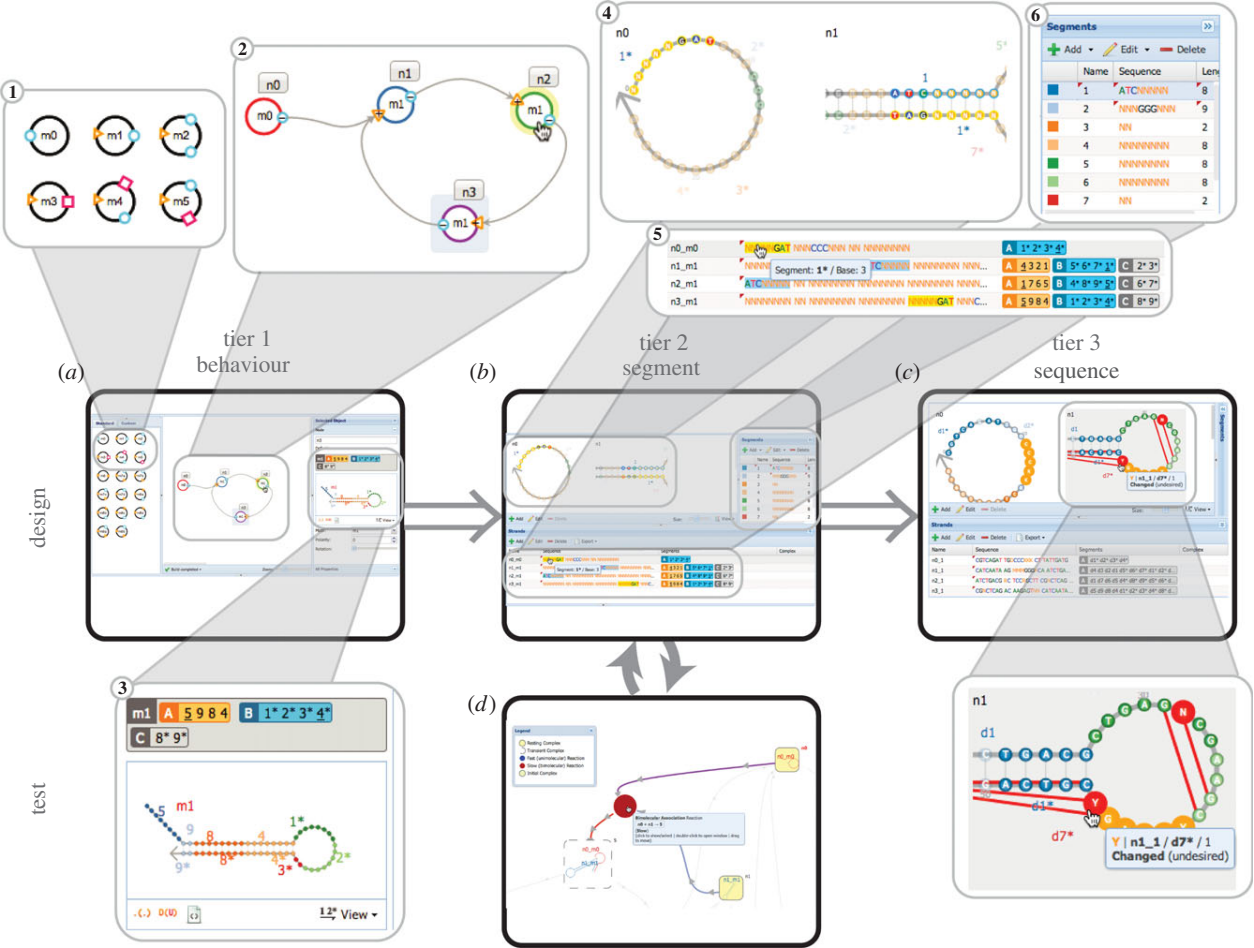
DyNAMiC Workbench screenshots. (High-resolution image—view PDF for details.) (*a*) Nodal (tier 1) design interface. Systems are composed by dragging-and-dropping nodes from the palette on the left **1**, then connecting nodes in the centre panel **2**. The right panel shows a preview of the molecular implementation **3**. See electronic supplementary material, figure S1, for details. (*b*) Segment-level (tier 2) design interface. Centre panel shows secondary structure view of each complex in the system **4**. Lower panel lists sequences and composition of strands in the system **5**. Right panel shows name and sequence of each distinct segment in the system **6**. See electronic supplementary material, figure S2, for details. (*c*) Multisubjective sequence (tier 3) design interface. Similar to the tier 2 design interface, different panels show complexes, strands and segments. The secondary structure view also highlights unintended interactions and shows bases flagged for modification by the analysis. See electronic supplementary material, figure S3, for details and images of other sequence design interfaces. (*d*) Reaction enumerator interface. Rectangular nodes represent complexes, joined by circular nodes representing reactions between intermediates. See figure 5 for details.

We have developed and unified graphical tools for designing and analysing systems at the three tiers of abstraction. Figure 1 describes the various tools we have implemented and/or integrated, and several tools that could be implemented in the future, as well as the intended workflow for a user developing a system with DyNAMiC Workbench. This section describes the input to, computation performed by, and output from each of these tools in detail, as well as discussing important features of the tools and their interfaces. Figure 4 shows screenshots of various tools.

### 3.1. Tier 1: behavioural/Nodal design

As described above, the Nodal formalism maps abstract behavioural units (node types) to known molecular implementations. The input to the Nodal compiler is a set of *instances* (Nodal species) of these node types, along with a prescribed connectivity between the ports of Nodal species. Input may be constructed by a ‘drag-and-drop’ graphical interface (figure 4*a* and electronic supplementary material, figure S1), or by describing the system using a text-based language called DyNAML (the *Dy*namic *N*ucleic *A*cid *M*arkup *L*anguage) [46]. The hairpin monomers and the single-stranded initiator described above (§2) are two examples of molecule types. In the example of the previous section, there were three distinct ‘species’ of the hairpin monomer molecule type. Like the three distinct hairpin monomers, each corresponding Nodal species will have a unique identity, but will share the same basic shape with the other species of the same type. In the same way, generated molecular species of the same type will have different sequences but similar dimensions and secondary structures.

The Nodal compiler uses the definition of each node type (the mapping to a known molecular implementation), as well as the network of connections between Nodal species, to produce a segment-level representation of the system—such that connections between nodes are implemented as appropriate Watson–Crick complementarity relationships between segments. To do this, the Nodal compiler first creates an instance of each molecular species (one for each Nodal species in the system), then labels the segments in the molecular species such that they satisfy the prescribed connections between nodes. The compiler begins with an initiator node, then traverses the network of connected nodes in a breadth-first search until all segments are labelled. The compiler will return an error if two complementarity statements are in conflict and cannot be fulfilled (e.g. if one connection requires a segment be labelled 7 and another connection requires that it be labelled 7*), or if a complementarity statement exists between domains of incompatible shape (e.g. if a domain A, comprising an 8-nt segment, is to be complementary to a domain B which contains an 8-nt segment and a 2-nt segment).

The output from the Nodal compiler is a segment-level (tier 2) representation of the system, encoded in the DyNAML Intermediate Language (DIL) [46]. This representation can be further edited by the user or converted to a sequence-level (tier 3) representation.

The Nodal language and compiler are highly general—rather than enforcing specific invariants on the form or structure of the underlying molecular primitives, or supporting only specific, pre-defined molecular motifs, our compiler allows the inclusion of systems with arbitrary nucleic acid secondary structures and behaviours. This flexibility is essential to supporting the previously discussed hairpin motifs, as well as more complex multi-stranded motifs (such as branched junctions)—both of which are important for structure formation applications. The language also allows arbitrary new motifs to be developed. DyNAMiC Workbench contains a collection of built-in node types with DNA implementations based on published literature (electronic supplementary material, figure S4), but also supports definition of new node types. A new node type can be defined by the following procedure: the segment-wise primary and secondary structure of the underlying molecule type are described, the molecule's segments are grouped into domains and the domains are assigned functional roles such as ‘input’ and ‘output’. New node types can also be defined from portions of existing systems. The ability to create ‘composite node types’ allows designed systems to be extended and re-used, by easily abstracting portions of an existing system into reusable components.

The software environment is built to be intuitive and easily usable, but also to provide detailed information to advanced users if necessary. A graphical interface (electronic supplementary material, figure S1*a*) reduces the barrier to entry for those unfamiliar with computer programming. Users construct Nodal species by dragging and dropping from a palette of pre-defined and custom node types. New node types with new molecular implementations can be defined by using a graphical interface (electronic supplementary material, figure S1*b*) or by selecting components of a Nodal program and exposing relevant domains on the nodes (electronic supplementary material, figure S1). For example, to define a reusable three-arm junction node type, the user could first define a three-arm junction system by composing nodes, then ‘wrap’ that system to define a new motif, exposing one input port. New node types can also be defined using DyNAML. The interface provides real-time feedback, generating interactive previews of the compiled molecular species and highlighting errors (as described above).

The Nodal language, compiler and design interface are described in detail in [46].

### 3.2. Tier 2: segment design

A tier 2 design comprises a description of each of the strands in a system, a division of the strands into segments, a statement of which segments should be complementary (or equal) to one another and an intended initial secondary structure for each of the strands. Essentially, a tier 2 design captures all of the desired inter-molecular and intra-molecular interactions between species in the system without assigning specific nucleotide sequences to segments. If the user has described their initial behavioural design using the Nodal formalism, DyNAMiC Workbench can automatically generate a tier 2 design as discussed above. The user may also choose to begin the design process at the segment tier and use DyNAMiC Workbench primarily for sequence design (§3.3); in this case, the user can input a tier 2 design directly using a graphical interface (figure 4*b*), or using a number of standard text-based input formats.

DyNAMiC Workbench uses the DIL as a common intermediate format for the tier 2 design; a graphical interface allows the user to modify the design at this tier and to transfer the design to various other tools (electronic supplementary material, figure S2). The user may change the number of nucleotides in existing segments, or impose specific constraints on the segments to be generated in tier 3. The user may inspect and modify the primary and secondary structures of the complexes in the system. The user may send the tier 2 design to *in silico* analysis tools, such as a segment-level reaction enumerator (§3.4.1). Similarly, the tier 2 design may be transferred to a sequence designer (tier 3) to generate specific nucleotide sequences that implement the design; this process is described next.

### 3.3. Tier 3: sequence design

The input to each of the ‘sequence designers’ is a segment-level (tier 2) representation of the system, along with any constraints or restrictions specified by the user (for instance, the user may want to hold the sequence of some particular segment fixed in order to incorporate a restriction enzyme cutting site).

Sequence design is the process of generating sequences of nucleotides to implement a particular set of complementarity and orthogonality relationships between nucleic acid strands. Several sequence design methodologies exist [27]. One approach uses calculations of the partition function [47–50] for an ensemble based on a detailed thermodynamic model of nucleic acid secondary structure [51]. This method attempts to maximize the probability that the ensemble adopts the intended secondary structure(s), while minimizing the chance that unintended interactions occur [27]. Recent work has extended this paradigm to entire test tubes of complexes [52]. It should be noted that these thermodynamic designers do not explicitly consider kinetic behaviour and that sequences with desirable thermodynamics may have undesirable kinetic properties [29]. One objective of future work is to explicitly incorporate kinetic models in sequence design.

Earlier methods focused on minimizing sub-sequence repeats or maintaining a minimum edit distance between non-complementary sequences in an ensemble—an approach known as ‘sequence symmetry minimization’ [26,53]. Evolutions of this approach [28,29] combine sequence symmetry minimization with some insights from thermodynamic models, as well as various heuristic methods, to produce sequences that meet additional criteria desirable for dynamic systems. These recent heuristic approaches may have favourable computational complexity compared to the thermodynamic approaches (scaling quadratically with the number of segments or strands, rather than cubically with the number of bases), but the actual computation time depends on the algorithm and the implementation. Finally, recent software combines these two approaches by passing limited subsets of a system between various designers—while independently analysing sequences according to a variety of criteria (e.g. attempting to explicitly eliminate secondary structure in key ‘toehold’ regions) [45].

Rather than selecting a single design methodology, our software incorporates and provides interfaces to various software tools that implement these different approaches (figure 4*c* and electronic supplementary material, figure S3). First, we have extended the core algorithms of Zhang's DD package, which performs heuristic, ‘segment-based sequence design’ [29], to add flexible stopping conditions and increase sequence diversity. Segment-based sequence design uses an approach similar to sequence symmetry minimization to design individual segments, which are then ‘threaded’ together to form full strands. In DD, a scoring function/hill-climbing algorithm is used to combine this technique with heuristic metrics of features believed to be important for dynamic systems. We have also developed a graphical interface to DD (electronic supplementary material, figure S3*a*). This interface allows the user to graphically add, remove and edit segments, as well as to thread these segments into strands. The individual segments and the full design can be visualized in real time as DD automatically tunes segments.

Additionally, we provide a direct interface to the NUPACK multi-objective thermodynamic sequence design [25] Web service (electronic supplementary material, figure S3*b*). Several tools in our package generate input scripts for the NUPACK designer, which can then be readily submitted to the NUPACK Web server.

Finally, we have developed an interface to the ‘Multisubjective’ sequence design package [45] (electronic supplementary material, figure S3*c*). Multisubjective uses several sequence design packages (the NUPACK multi-objective designer and DD) to eliminate unintended secondary structure from key regions of strands in dynamic systems. This is an iterative process—sequence design is initially performed with another sequence designer (DD or NUPACK), then sequences are evaluated by Multisubjective. Multisubjective identifies unintended secondary structure, long regions of repeated nucleotides, etc. and proposes mutations to the designed sequences. The sequences, with mutations, are then re-submitted to the primary sequence designer and the process is repeated. Our interface allows the user to inspect the analysis performed by Multisubjective and edit the suggested modifications before redesigning.

We note briefly that, though our focus is on DNA, the design considerations are essentially identical for RNA (though thermodynamic designers will use different sets of parameters [51,54,55]). All three packages discussed also support the design of RNA sequences. Design and evaluation of mixed DNA/RNA sequences is outside the scope of current work.

The output of each of these sequence designers is a set of nucleotide sequences for each of the complete strands in the system. These sequences can be exported to a text file or a spreadsheet, which can be used to order sequences from a commercial oligonucleotide synthesis provider.

### 3.4. Analysis tools

#### 3.4.1. Enumerator

Reaction enumeration is the process of predicting the network of possible reactions between a given set of starting complexes. For dynamic nucleic acid systems, a reaction enumeration at the level of segments (rather than individual nucleotides) can provide valuable insights into the behaviour of the system (by culling much of the detail of a sequence-level enumeration and focusing on larger interactions). Results of a segment-level enumeration could also be used to perform stochastic or analytical mass-action kinetic simulations of the reaction networks in order to predict physical behaviours of systems *in vitro*. Finally, segment-level reaction enumeration could be used for formal verification of system behaviour.

**Figure 5.**
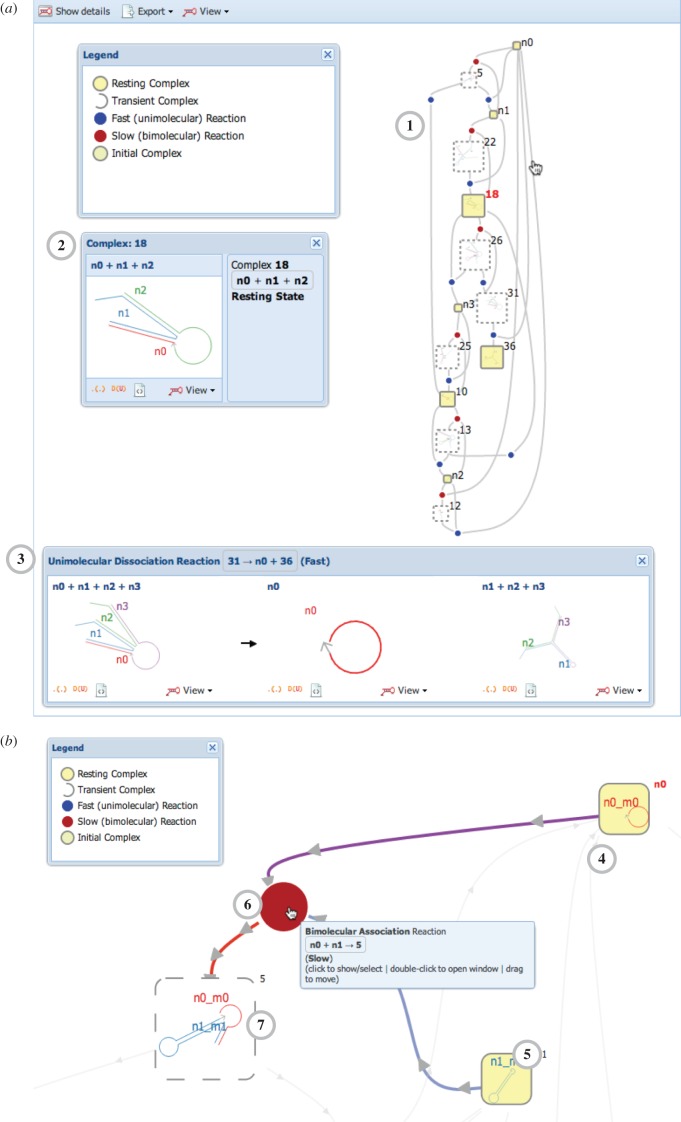
Graphical interface to a reaction enumerator [56]. (High-resolution image—view PDF for details.) (*a*) The enumerator calculates a network of possible reactions **1** between the starting complexes, as well as the possible intermediate complexes formed. The user can pan and zoom throughout the network to view intermediate complexes. Double-clicking a complex **2** or reaction **3** allows its structure to be inspected. The reaction network shown here is the execution of the three-arm junction system—a detailed version of the schematic in figure 3*h*. (*b*) The user can hover over components of the graph to highlight connections to neighbouring components—in this case, arrows showing the reactants **4**, **5** of a bimolecular association reaction (red dot, **6**) are highlighted in blue and purple, while the arrow showing the product **7** is highlighted in red. Additionally, the graph layout can be re-arranged by dragging-and-dropping complexes, reactions and arrows. These interactive features allow larger reaction graphs to be explored and interpreted.

We have built an interface for performing segment-level reaction enumeration [56] and visualizing the results (figures 4*d* and 5). Our interface displays a graph of starting, intermediate and resting/end-state complexes, connected by the reactions between these complexes. The interface automatically colours fast versus slow reactions, transient/short-lived versus resting/long-lived complexes and initial complexes versus enumerated complexes. The graph can be readily traversed by zooming and panning, and the interface highlights relevant substrates or reactions proximal to a complex of interest (for instance, all reactions producing or consuming a given complex can be highlighted). The graph can be re-arranged by dragging and dropping to aid visualization. Each complex or reaction can also be isolated and visualized as an interactive secondary structure diagram.

The reaction enumerator is described in greater detail in ref. [56], as well as in an upcoming publication.

#### 3.4.2. Sequence-level analysis

Once system-level behaviour has been designed and analysed, and nucleotide sequences have been generated, sequences can be analysed to determine experimental suitability. A variety of analyses can be performed. For small self-assembly systems, the MFE structure and base pair probabilities of the entire ensemble can be determined using a full physical model which computes the partition function for the system [49,50]. For larger dynamic systems (beyond tens of species or hundreds of bases), this exhaustive computation becomes impractical. However, the MFE structure and base pair probabilities of individual strands or small subsets of strands may still be examined to discover unintended secondary structure within strands or spurious interactions between strands.

Many packages exist which can perform these computations. Our software integrates directly with several publicly available Web services for sequence-level analysis. The NUPACK Web server can perform full partition function and pair probability calculation [25]. The Mfold and DINAmelt Web servers provide several types of analysis [31,38]. The TBI Vienna RNAfold Web server allows computation of the MFE structure and partition function [32]. These software tools provide a wide range of options and allow the use of various available thermodynamic parameter sets for both DNA and RNA. DyNAMiC Workbench allows users to easily submit any sequence displayed within the software to these Web servers for analysis through a simple, unified interface.

### 3.5. Utilities

#### 3.5.1. Structure visualization

DyNAMiC Workbench includes a flexible system for visualizing arbitrary unpseudoknotted nucleic acid secondary structures, which was used to generate all images of secondary structure that appear in this paper. The basic visualization combines a traditional tree-based ‘planar graph’ [57,58] layout with a linear representation (allowing branched structures to be more easily visualized) and an interactive colouring scheme. This visualization is used throughout the software to display secondary structures, and can also be accessed as a separate utility.

#### 3.5.2. Sequence manipulation

Finally, a suite of utilities is provided in DyNAMiC Workbench to perform various common transformations on nucleic acid sequence strings. These range from Watson–Crick complementation to Levenshtein distance calculation [59] to threading segments into strands. The user can also quickly perform sequence-level thermodynamic analysis (through the interfaces described above). Finally, sequences can be exported to/imported from various standard formats (FASTA, CSV, etc.) These utilities are available in context anywhere the user may interact with sequences.

## 4. Architecture

**Figure 6.**
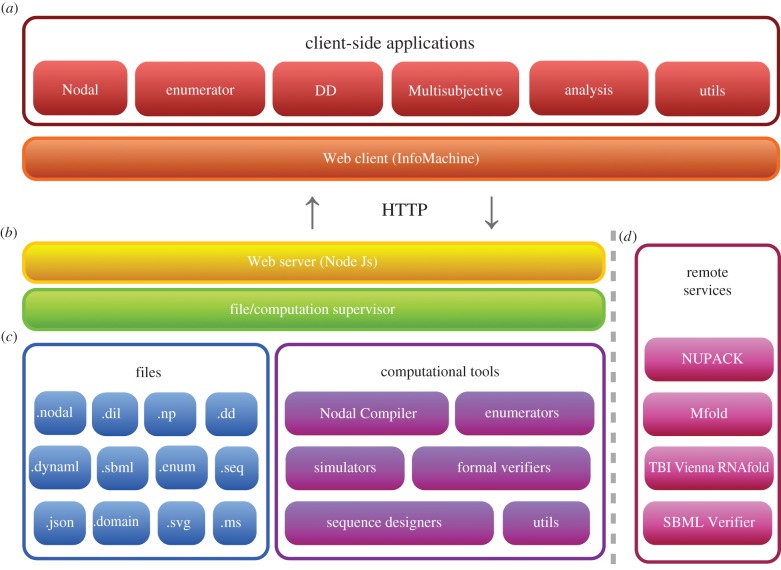
Software architecture. (*a*) The architecture supports a rich set of high-level, client-side applications. These applications are presented graphically to the user. (*b*) A Web server exposes this interface and handles requests from the client-side applications, dispatching those requests to the computational tools and the file system. (*c*) A wide variety of file formats are supported, and many conversions between related formats can be performed automatically. These files are then consumed by computational tools, which may run on the same machine as the Web server/supervisor, or be located remotely (e.g. run on a cluster). (*d*) The Web interface interacts directly with tools that are provided independently as Web services. For instance, the Web interface is capable of submitting requests directly to various sequence analysis servers [25,31,32,38].

The software is deployed as a Web service—software tools are installed on a server and managed by a supervisor layer, which is tightly connected to a Web server (figure 6). The Web server exposes a rich client-side interface. The supervisor manages user files, converting between different formats and launching computational tools. Examples of server-side computational tools include the Nodal compiler and the segment-level enumerator. The core Web server and supervisor layers are written in JavaScript and are executed by NodeJS—an evented, asynchronous runtime and Web server based on Google's V8 JavaScript engine.

A benefit of the client–server architecture is that server-side tools can be written in any language, compiled or deployed for a single architecture (that of the server), and made available to clients running on many platforms through the Web interface. Various server tools are currently written in JavaScript, Python, C and C++.

The client-side interface comprises a file manager and many applications that provide graphical interfaces to the underlying server tools. Examples of client-side applications include the Nodal designer, DD and the enumerator interface. The client-side interface is written in JavaScript and uses a variety of open-source user interface toolkits. User registration allows designs and other user files to be saved on the server across multiple sessions.

## 5. Discussion

We have presented software that facilitates a design process that is hierarchical and iterative. A user designs an abstract behaviour which can be compiled into segments and then sequences. The ability to easily enter the design process, to perform analysis, and to make edits to the design at any tier of abstraction (behavioural, segment or sequence) makes DyNAMiC Workbench an effective platform for iterative design. The goal of this flexible design process is to allow for fewer experimental design iterations, by moving *in silico* much of the testing and ‘debugging’ that would have been done by costly and time-consuming laboratory experiments. We have shown an example system designed using the software, and we present four additional designs in the electronic supplementary material—a larger, six-arm junction (electronic supplementary material, §S2.1); a bounded dendrimeric structure (electronic supplementary material, §S2.2); an exponentially-amplifying catalytic circuit (electronic supplementary material, §S2.3); and a self-assembling three-dimensional tetrahedron (electronic supplementary material, §S2.4). With these designs, we discuss in greater detail the practical challenges of implementing large systems using the software.

We have chosen to incorporate several existing tools for each stage of the design process. For instance, when designing sequences, users can easily choose one of the several tools discussed above, or can combine the results of several tools. The availability and interoperability between multiple tools, even for the same task, provides two benefits. First, different tools may be better suited to perform a given task under different circumstances; for instance, DD may be more useful for design of large kinetic systems, while a thermodynamic designer such as NUPACK may provide a more rigorous design for smaller systems. Second, easy rapid switching between different tools will allow for better comparison of tools' performance and improved characterization of tools' strengths and weaknesses for different tasks.

Our software is deployed as a publicly available Web service. This design decision was motivated by several observations. First, installation of software (especially software which requires the user to build an executable from source code, or to use the command line) can be a significant barrier to adoption. Second, use within the field of a variety of file formats can require tedious manual juggling when working in traditional command-line or desktop interfaces. Third, many software tools in the field require significant computational resources; providing these tools as a Web service allows users to use or at least experiment with these tools without making the significant upfront investment in computational resources. The Web-based interface allows the user to access a graphical interface to the service, using any device with a modern Web browser. This makes the service truly cross-platform and also obviates the need for the user to install any software or to maintain an updated version of the software—updates to both the interface and the tools themselves can be centrally managed. For users who manage their own research computing facilities, the software is also available as a downloadable package.

The software has been designed with extensibility in mind; while we have worked to integrate many existing tools, this is only a starting point. Many excellent packages exist which have not yet been integrated. To this end, the architecture allows new server-side tools to be added and new client-side applications to be easily developed. We intend this software and the associated service to serve as a ‘clearinghouse’ for dynamic DNA/RNA nanotechnology software development and deployment. We have taken several steps towards this goal: first, the entire source code for the project is to be released under the GNU General Public License. We hope this will encourage users to modify and contribute to the software, in addition to encouraging usage of the public Web server. Second, we have developed and provided extensive documentation of the application programming interface (API) for the software, to facilitate development of third party extensions; this API documentation is linked to from within DyNAMiC Workbench's built-in user manual, and can be accessed from the main ‘Help’ page within the software.

As summarized in table 1, there are many possible features that are yet to be implemented. Reaction enumeration could be performed at the behavioural tier, so that the intended reaction pathway can be compared directly to the expected reaction pathway at the segment tier. The behavioural- and segment-tier reaction enumerations could also be used to perform kinetic simulations and determine the time-evolution of concentrations of intermediate complexes. Electronic supplementary material, figure S5, demonstrates some of these possible extensions.

In future work, we intend to improve capabilities for *in silico* analysis, as well as provide higher level design tools for self-assembling systems. Specifically, we intend to incorporate various simulation tools based on the segment-level enumerator—for instance, ODE-based or stochastic kinetic simulators—as well as separate, base-level kinetic simulation tools [39]. It would be interesting to develop tools for higher level design of self-assembly processes; for instance, a two- or three-dimensional ‘molecular canvas’, which would eventually allow users to quickly and easily translate two-dimensional images and three-dimensional structures to molecular implementations. Expanding individual tools to better handle more diverse structural motifs (for instance, pseudoknots, which are currently prohibited by the compiler and enumerator) and reaction types is another area for future work. Finally, in the Nodal compiler included with DyNAMiC Workbench, there is a one-to-one mapping between pre-defined node types and corresponding molecular implementations. However, this need not be the case for every behavioural designer. For instance, a future behavioural designer could operate on abstract chemical reaction networks, adopting one of several translation schemes [60,61] to convert the behaviour into a molecular structure. We anticipate many such future designers are possible.

Finally, an ongoing goal will also be to seek out and integrate valuable existing tools; we hope this will be a collaborative effort embraced by the entire community.

## Supplementary Material

Supplementary Information
