# Supplementary material for: DyNAMiC Workbench: an integrated development environment for dynamic DNA nanotechnology
Source: J R Soc Interface. 2015 Oct 6;12(111):20150580. doi: 10.1098/rsif.2015.0580 (PMC4614494; doi:10.1098/rsif.2015.0580)
Supplement: Supplementary Information [file rsif20150580supp1.pdf]

Supplemental Information for: DyNAMiC Workbench: An Integrated Development Environment for Dynamic DNA Nanotechnology

Casey Grun* [1], Justin Werfel[1], David Yu Zhang[1,3], and Peng Yin†[1,2]

[1] *Wyss Institute for Biologically-Inspired Engineering, Harvard University*
[2] *Department of Systems Biology, Harvard Medical School*
[3] *Present Address: Department of Bioengineering, Rice University*

Contents

S1 Supplemental Figures 4

S2 Supplemental Design Examples 8
- S2.1 Six-arm junction . . . 10
- S2.2 Bounded Dendrimer . . . 18
- S2.3 Catalytic Amplifier . . . 28
- S2.4 Tetrahedron . . . 33
- S2.5 Discussion . . . 41

List of Figures

- S1 Nodal (tier 1) design interface . . . 4
- S2 Segment-level (tier 2) design interface . . . 5
- S3 Sequence (tier 3) design interface . . . 6
- S4 Built-in node types . . . 7
- S5 Future extensions . . . 8
- S6 Example systems from the literature . . . 9
- S7 Nodal representation of 6-arm junction . . . 10
- S8 Segment-tier representation of 6-arm junction . . . 11
- S9 Full reaction enumeration for 6-arm junction . . . 12
- S10 Condensed reaction enumeration for 6-arm junction . . . 13
- S11 Sequence-tier representation of 6-arm junction . . . 16
- S12 Thermodynamic analysis of DD-designed sequences for 6-arm junction . . . 17
- S13 Nodal representation of bounded dendrimer . . . 18
- S14 Segment-tier representation of bounded dendrimer . . . 19
- S15 Full reaction enumeration for bounded dendrimer . . . 21
- S16 Full reaction graph for bounded dendrimer, highlighting monomers `A3` and `B3` . . . 22
- S17 Condensed reaction enumeration for bounded dendrimer . . . 23
- S18 Condensed reaction enumeration for bounded dendrimer highlighting paths to final product . 24
- S19 Sequence-tier representation of bounded dendrimer . . . 26

*`casey.grun@wyss.harvard.edu`
†`py@hms.harvard.edu`

S20 Thermodynamic analysis of DD-designed sequences for the bounded dendrimer 27
S21 Nodal representation of catalytic amplifier 28
S22 Segment-tier representation of catalytic amplifier 29
S23 Full reaction enumeration for catalytic amplifier 30
S24 Condensed reaction enumeration for catalytic amplifier 31
S25 Sequence-tier representation of catalytic amplifier 32
S26 Thermodynamic analysis of DD-designed sequences for the catalytic amplifier 32
S27 Nodal representation of tetrahedron 33
S28 Segment-tier representation of tetrahedron 34
S29 Full reaction enumeration for tetrahedron 35
S30 Condensed reaction enumeration for tetrahedron 36
S31 Full reaction enumeration for tetrahedron, highlighting reactions involving `n8`, `n9`, and `n10` . 37
S32 Sequence-tier representation of tetrahedron 39
S33 Thermodynamic analysis of DD-designed sequences for the tetrahedron 40

S1 Supplemental Figures

a)

b)

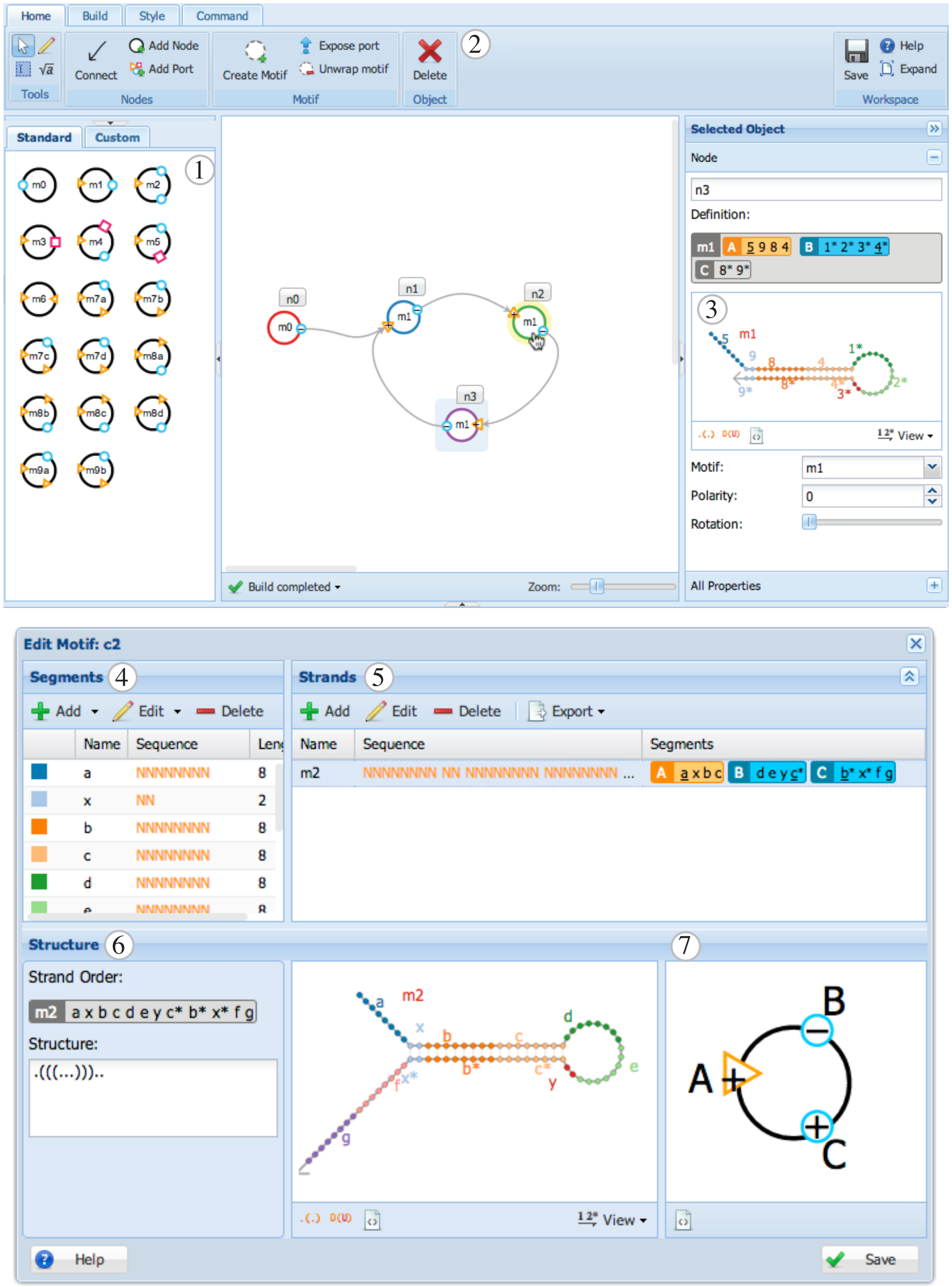

Figure S1: Nodal (tier 1) design interface. **(a)** Users may drag built-in or custom node types from the palette on the left ①. The ribbon atop the screen allows the user to select elements, construct new nodes or node types, and prescribe complementarity relationships between ports ②. Users can also insert text, drawings, and mathematical formulae as annotations. Selecting nodes shows a preview of their molecular implementation in the compiled system ③. Systems are continuously compiled in real-time, and computed properties such as domain polarities are updated to reflect changes as the system is designed. Using the Build tab, users can generate and view segment-level (tier 2) designs. **(b)** Users may define new node types from *de novo* molecular architectures using a graphical interface. Segments ④ and then strands ⑤ are defined and composed together into complexes ⑥, which are labeled to produce node types ⑦.

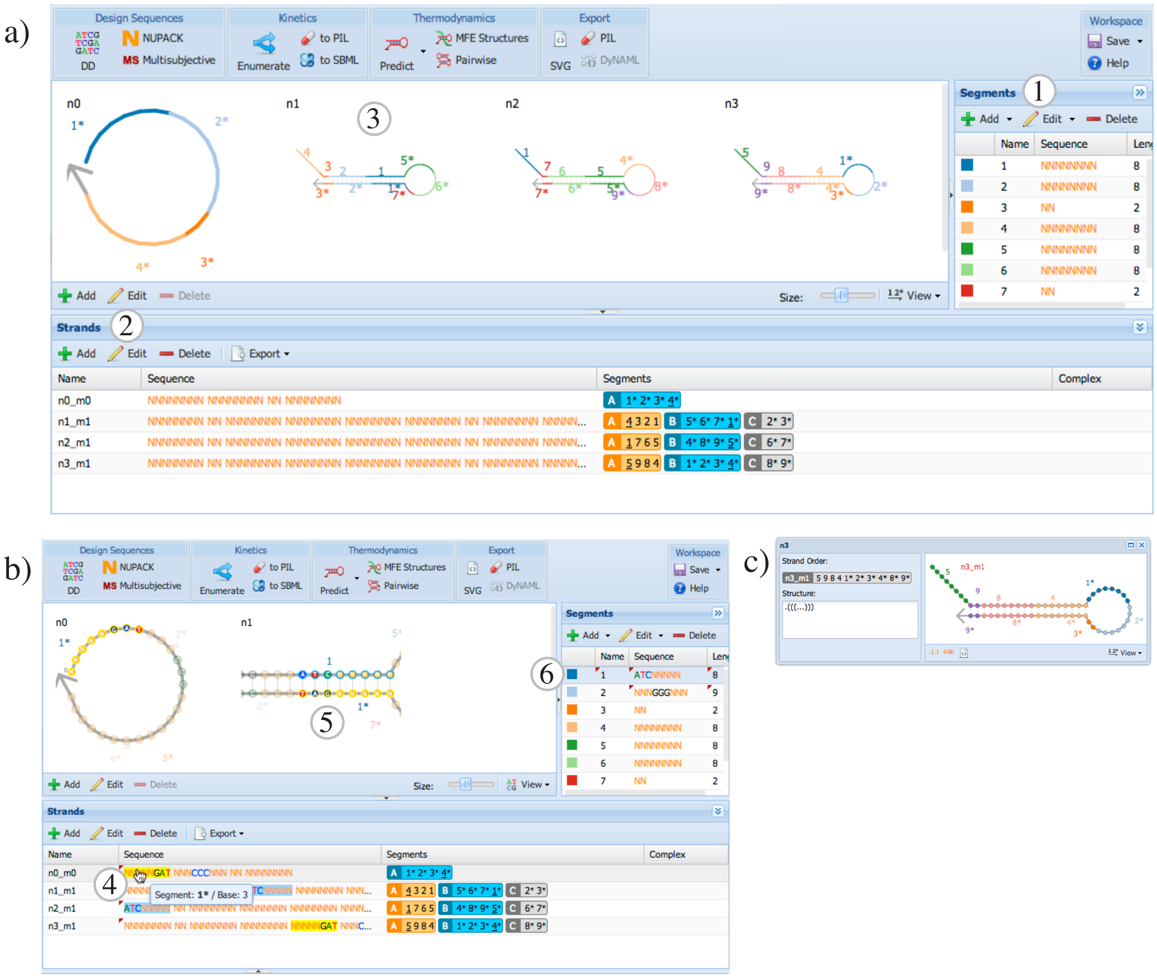

Figure S2: Segment-level (tier 2) design interface. **(a)** Users may view a preview of each of the segments ①, strands ②, and complexes ③ generated from the Nodal-level description. Segments, strands, and complexes may be edited in this view. Sequence constraints can be added to the design, or previously-designed sequences can be visualized on the structure. Individual bases in segments may be colored by segment identity, domain role (as in the Nodal view), or base identity (A, T, C, or G), and various view options may be specified. Segment-level designs may be exported to a variety of formats, or may be sent to sequence designers. Segment-level reaction enumeration, as well as sequence-level thermodynamic calculations, may be performed. **(b)** The user may point to segments ④ to highlight their appearance elsewhere in the design and interface ⑤, ⑥, making it easy to see where sequences (yellow) and their complements (light blue) propagate to other strands or species. **(c)** Complexes may be edited by selecting or re-arranging their constituent strands, or by modifying their secondary structure.

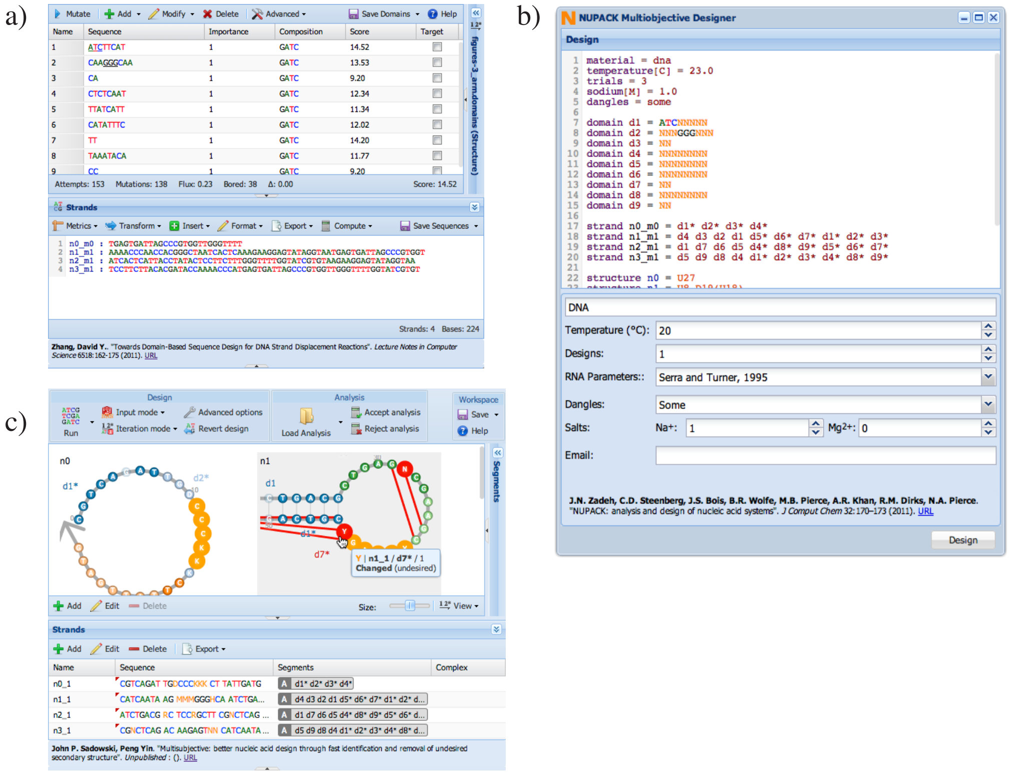

Figure S3: Sequence (tier 3) design interface. **(a)** Sequence design using the web interface to the sequence designer DD [1]. Sequence mutation can be started and stopped; sequences can be added, modified, and deleted; and designs can be saved from the toolbar atop the center pane. The right pane allows the sequences to be assembled into strands. The bottom pane allows users to view the final designed strands, as well as edit, format, and export the generated sequences to a variety of formats. **(b)** Users can also write and submit designs to the NUPACK Web Server [2] for multi-objective thermodynamic design. **(c)** Sequence design using the iterative, multi-paradigm sequence designer Multisubjective [3]. The interface allows the user to manage multiple iterations of a design, to view the results of Multisubjective's analysis, and to modify the design after each iteration. Undesired interactions are shown as red lines, while bases "frozen" as immutable are faded. Long prevented sequences (e.g. poly-G, poly-R, etc.) are also marked (not shown in this screenshot).

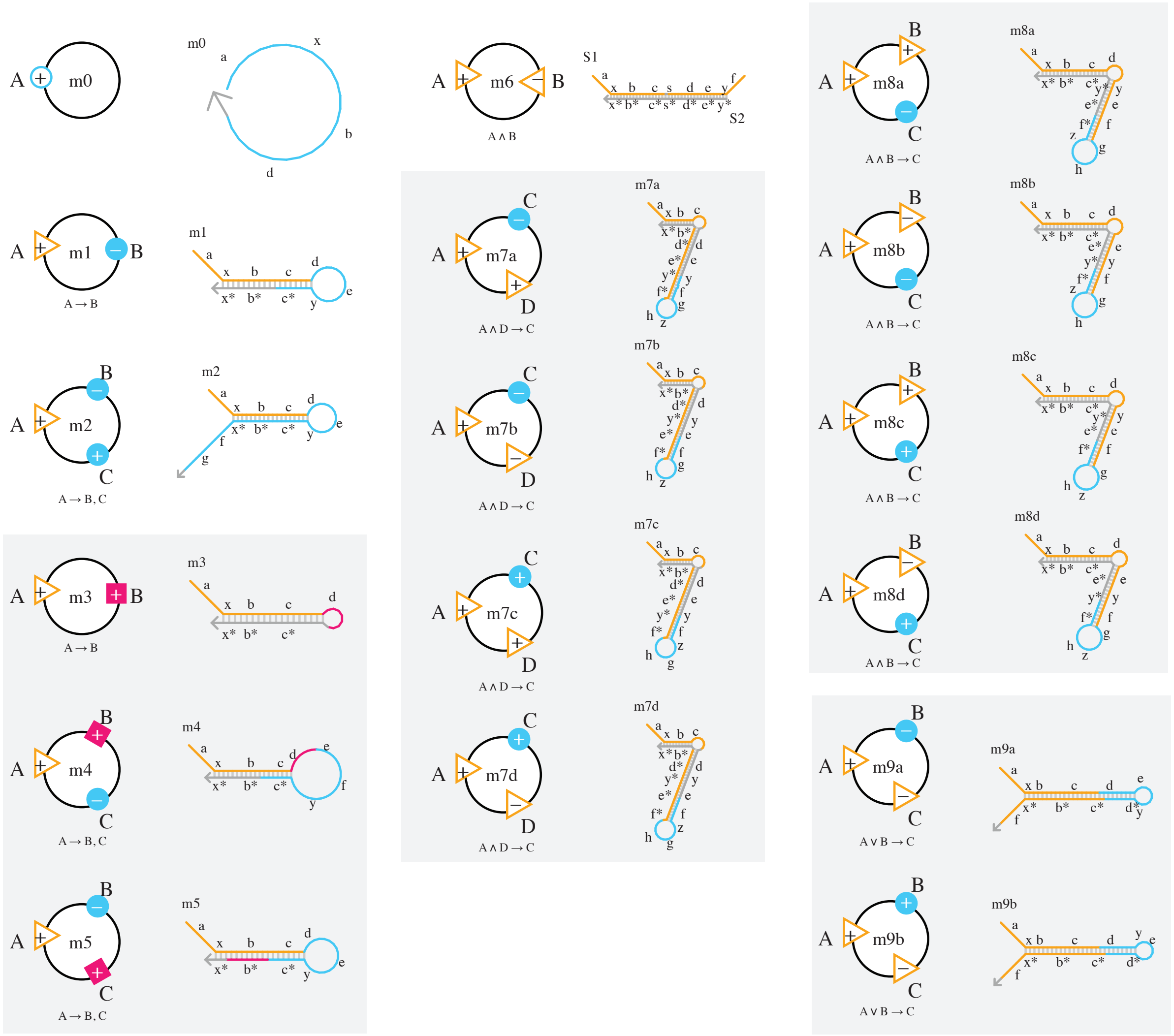

Figure S4: Built-in node types. These node types and their corresponding molecular implementation are bundled with DyNAMiC Workbench, and can be added to Nodal designs via a simple drag-and-drop mechanism. (Additional node types for specific scenarios can be created, as described in the text; we provide this set intended as a useful starting point based on our experience and existing literature). Node types are labeled with a lowercase 'm', a number, and a letter representing a particular variation (e.g. `m7a`, `m7b`, `m7c`, and `m7d` are all variations of a single shape `m7`). Triangular ports represent input domains; circular ports represent output domains, which are triggered by corresponding inputs. Square ports represent "bridge" domains which participate only in ring-closing reactions and do not perform branch migration. Node types `m0`–`m5` are simple input-output hairpins (some of which have special, ring-closing "bridge" domains, shown in pink), based on refs. [4, 5]; `m6` is a cooperative hybridization complex—it requires both inputs to be present in order for either to stay bound—based on ref. [6]; `m7a–d`, `m8a–d`, and `m9a–b` are boolean AND and OR gates, inspired by ref. [7] but not experimentally validated.

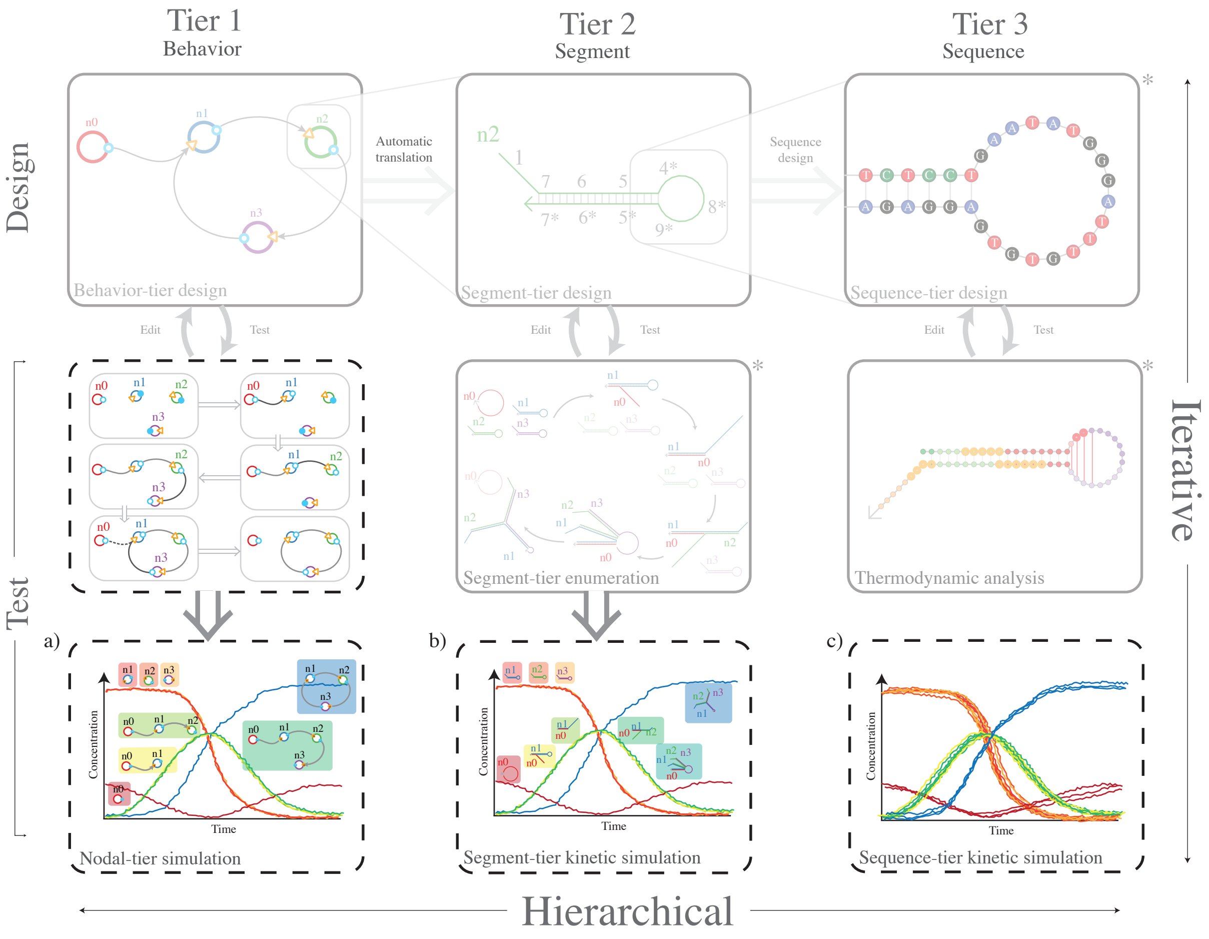

Figure S5: Future extensions. This figure describes areas for future work as an expanded version of Fig. 1. In addition to the implementation and analysis tools currently provided, tools for kinetic simulation are important areas for future work. Boxes with dashed outlines are not yet incorporated by DyNAMiC Workbench. Kinetic simulations may be performed at various tiers—in terms of **(a)** behavioral units, **(b)** segments or **(c)** individual nucleotides. These simulations could predict the relative prevalence of the possible reaction pathways and give estimates of time-course and steady-state concentrations of species. This simulation could be performed on a reaction network enumerated as described in Sec. 4.4.1, or the simulation could be performed alongside the enumeration (as in Visual DSD's "just in time" mode [8]).

S2 Supplemental Design Examples

In this supplemental section, we describe a set of example systems designed using DyNAMiC Workbench. Each of these systems has been demonstrated experimentally by other authors. [1] [4, 5, 9] Therefore these constitute plausible design problems that DyNAMiC Workbench could be used to address. For each system, we first describe the motivation for constructing that system, then present a design of the system at each of the three tiers: the behavioral (Nodal) tier, the segment tier, and the sequence tier. We provide a segment-tier enumeration of possible reactions for the system, as well as a thermodynamic evaluation of the designed sequences. Throughout the section, we will attempt to highlight some of the real-world issues encountered in designing these systems. Fig. S6 outlines the four example systems we will consider from the literature.

[1]The original authors did not implement the systems using DyNAMiC Workbench

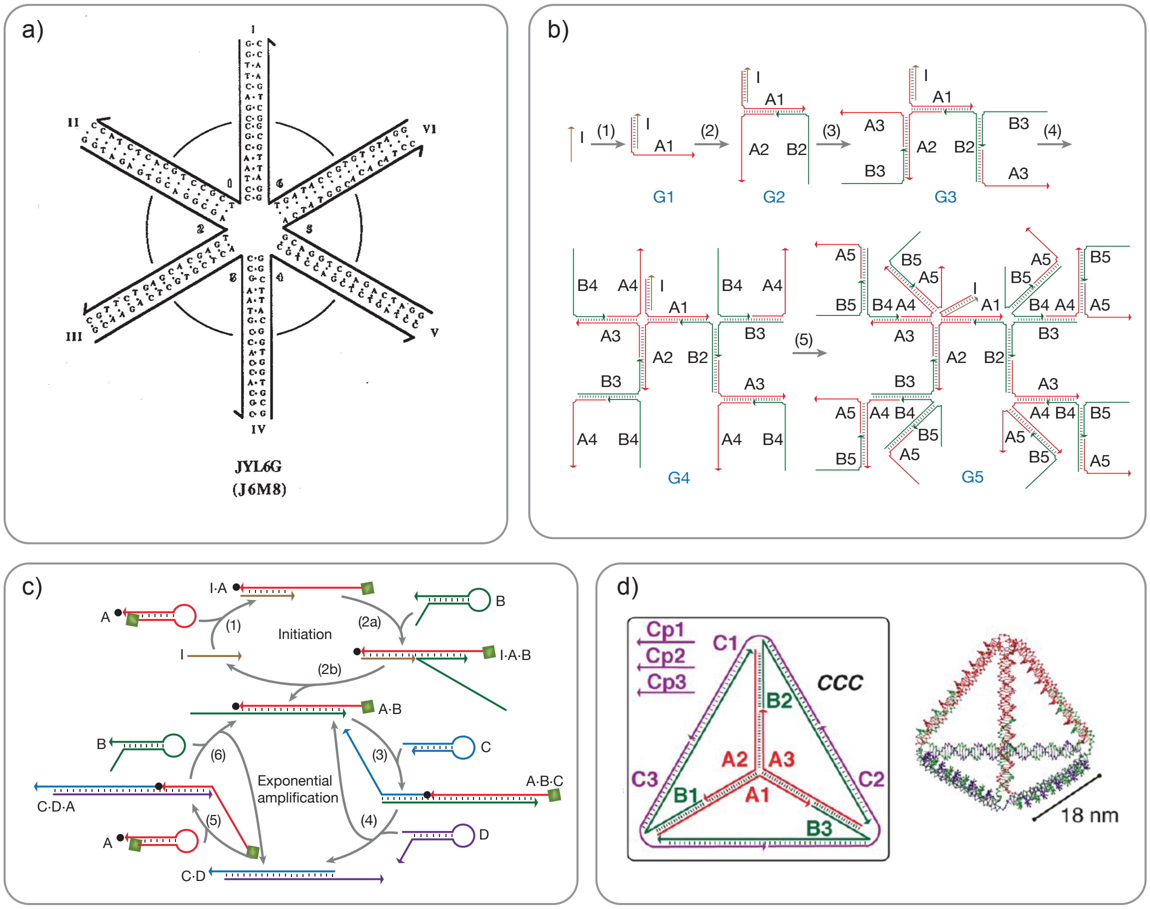

Figure S6: Example systems from the literature. **(a)** Six-arm junction. Originally demonstrated by Wang et al. [9], the junction shown in this figure is a generalization of Seeman's thermodynamically-directed immobile junctions [10, 11]. Our example will instead follow the "developmental self-assembly" approach of Yin et al. [4], and will thus appear as a generalization of our three-arm junction example from Sec. 3. Panel reproduced from [9], Fig. 1 (©1991 The American Chemical Society). **(b)** Bounded dendritic structure. Again, we will follow the developmental self-assembly paradigm in constructing a branched tree structure that exhibits triggered exponential growth in size. Panel adapted from [4], Fig. 4 (©2008 Nature Publishing Group). **(c)** Exponential amplification circuit. This system exhibits autocatalysis and exponential kinetics in the model of Turberfield et al.'s free-running machine [12], but uses hairpins for toehold sequestration. Panel reproduced from [4], Fig. 3 (©2008 Nature Publishing Group). **(d)** Kinetically-assembled DNA tetrahedron. This system demonstrates the ability of developmental self-assembly to produce complex, 3D topological structures. Panel adapted from [5], Fig. 2 (©2014 The American Chemical Society).

S2.1 Six-arm junction

Branched junction structures (Holliday junctions) from biological systems served as the original inspiration for the field of DNA nanotechnology [10]. In biology, these branched junctions are *mobile*, and they eventually resolve to form duplexes by enzymatic cleavage or branch migration. One of the earliest demonstrations [11] of DNA nanotechnology was the construction of an *immobile* junction that could not re-arrange or resolve by 4-way branch migration. Branched junctions of various kinds have served as the foundation for all major efforts in structural DNA nanotechnology [13]. Numerous structures have been constructed using branched junctions—cubes [14], octahedra [15], irregular graphs [16], and others. The potential connectivity of these large graph-like structures is limited by the number of "arms" in the branched junction—Holliday junctions in biological systems have four "arms;" however, junctions with 3, 5, 6, 8, and 12 arms have also been constructed synthetically [4,9,17]. Most of these structures were formed by thermal annealing—their kinetic assembly process was neither specified nor controlled. Similarly, these structures cannot be triggered by introduction of a catalytic initiator.

In Sec. 3 of the main text, we presented the scheme for construction of a three-arm junction structure based on [4] (Fig. 3); this structure was catalytic (an initiator species was introduced, but was released at the end of the assembly process), stepwise (strands in solution could only enter the assembly in a specific order, corresponding to the desired kinetic pathway), and, importantly, *triggered* (the monomer species making up the bulk of the structure would not self-assemble until the initiator was present). Here we present an extension of this scheme to the formation of a six-arm junction. An advantage of the Nodal architecture is that it allows the self-similar arms of the junction to be represented as duplicates of the same structural template—the same "node type" (`m1`). As a consequence, the program to generate the six-arm junction (Fig. S7) is a natural extension of that for the three-arm junction; we can follow the same pattern as before, beginning with an initiator node, which is then connected to a cycle of arms. The complementarity between the final node (`n7`) and the initial node (`n2`) allows the initiator to be displaced, prompting catalysis and closing the junction.

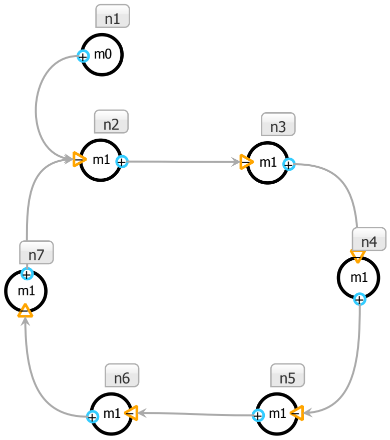

Figure S7: Nodal representation of 6-arm junction

The Nodal compiler automatically generates a segment-tier representation of the system (Fig. S8) based on the definitions of the built-in node types `m0` and `m1` (see Fig. S4 for the definition of the built-in node types included in DyNAMiC Workbench). One notable feature of the hairpin architecture is that the intra-strand complementarity constraints necessary to form the hairpin monomers result in a propagation of sequence complementarities thoughout the system. For instance, note that even though `n2` and `n4` should not directly interact, they have shared complementarity (5^* appears in `n2` and `5` appears in `n4`). This is not a serious problem, since `5` is only a (relatively short) toehold, and there are no adjacent recognition segments. Segment 5^* is also sequestered within the loop region in `n2`, meaning any branch migration mediated by 5^* as a toehold

would need to pass through an unfavorable, sterically-hindered, pseudoknotted intermediate (any binding to `5*` in `n2` would result in a pseudoknot). Nevertheless, careful consideration is necessary in other systems to avoid the unintentional propagation of these unintended constraints—this issue motivates the need to verify systems' behavior using a reaction enumerator.

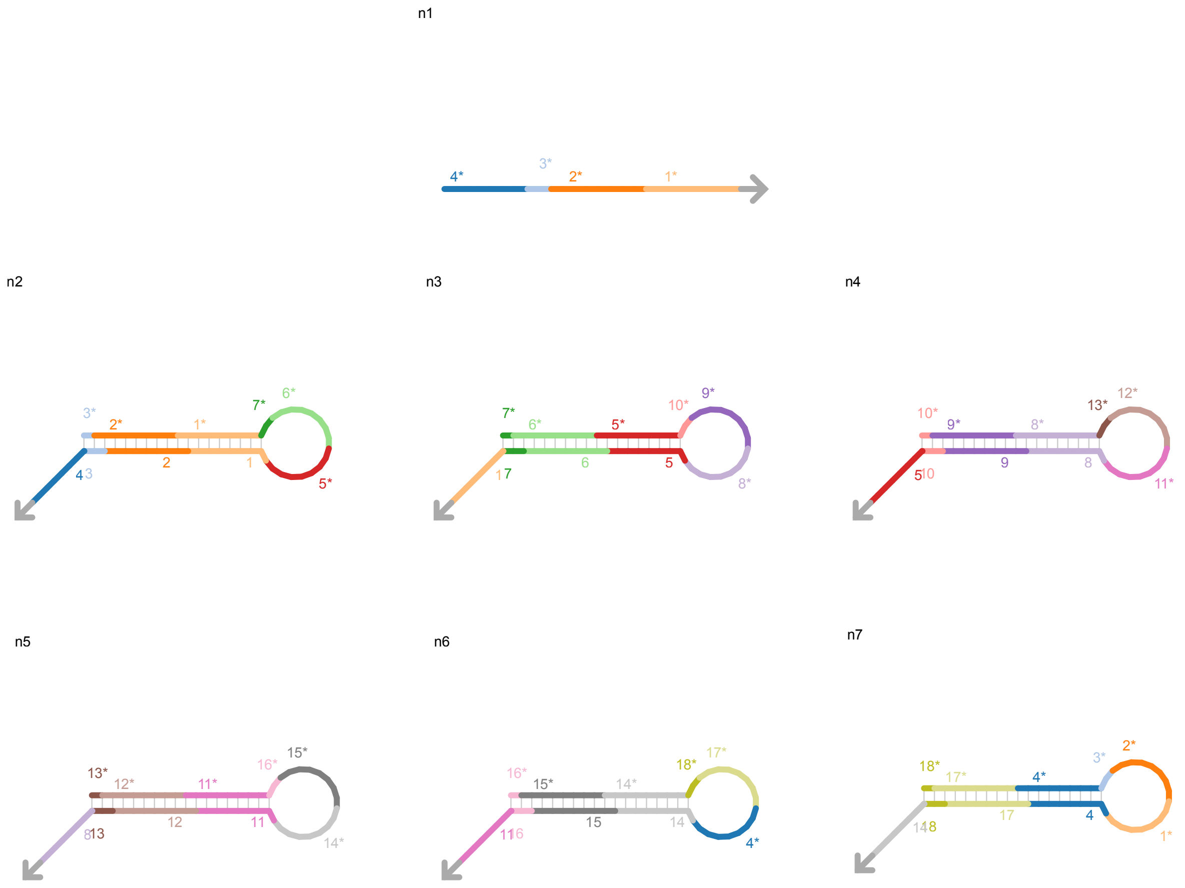

Figure S8: Segment-tier representation of 6-arm junction. Colors indicate distinct segment identities.

We can evaluate this segment-tier design using the reaction enumerator. The full network of reactions (Fig. S9) is relatively straightforward to interpret—the first hairpin (`n2`) is opened by the initiator (`n1`); then, each of the initial complexes `n3`–`n7` (shaded with a light-blue background) joins the growing structure, in order. Each of these associations happens in two steps. First, the toehold on the hairpin binds to an exposed segment on the growing structure in a bimolecular association reaction (red dot); this results in a transient complex (dashed outline) showing the toehold-bound nucleation step (for instance, complex `8`). Second, there is a fast unimolecular rearrangement (blue dot) which results in opening of the hairpin by branch migration. These two steps occur for each hairpin `n3`–`n7`. Finally, after `n7` has joined the structure, the last intermediate complex (complex `75`) undergoes a unimolecular rearrangement and dissociation reaction (via 3-way branch migration with a remote toehold), in which the last hairpin `n7` displaces the initiator `n1` to complete the junction structure (complex `80`).

The condensed reaction view (Fig. S10) shows essentially the same process, but none of the nucleation steps are shown (because the transient complexes resulting from nucleation are resolved to their resting state fates—see Sec. 4.4.1 for details). The condensed view allows one to clearly see the stepwise assembly process consisting of the sequential addition of the initial complexes to the growing structure (yellow boxes).

Once we have verified that the assembly process is expected to occur correctly (based on the segment-tier mode), we can design sequences to implement this program of complementarities. In this section, we will

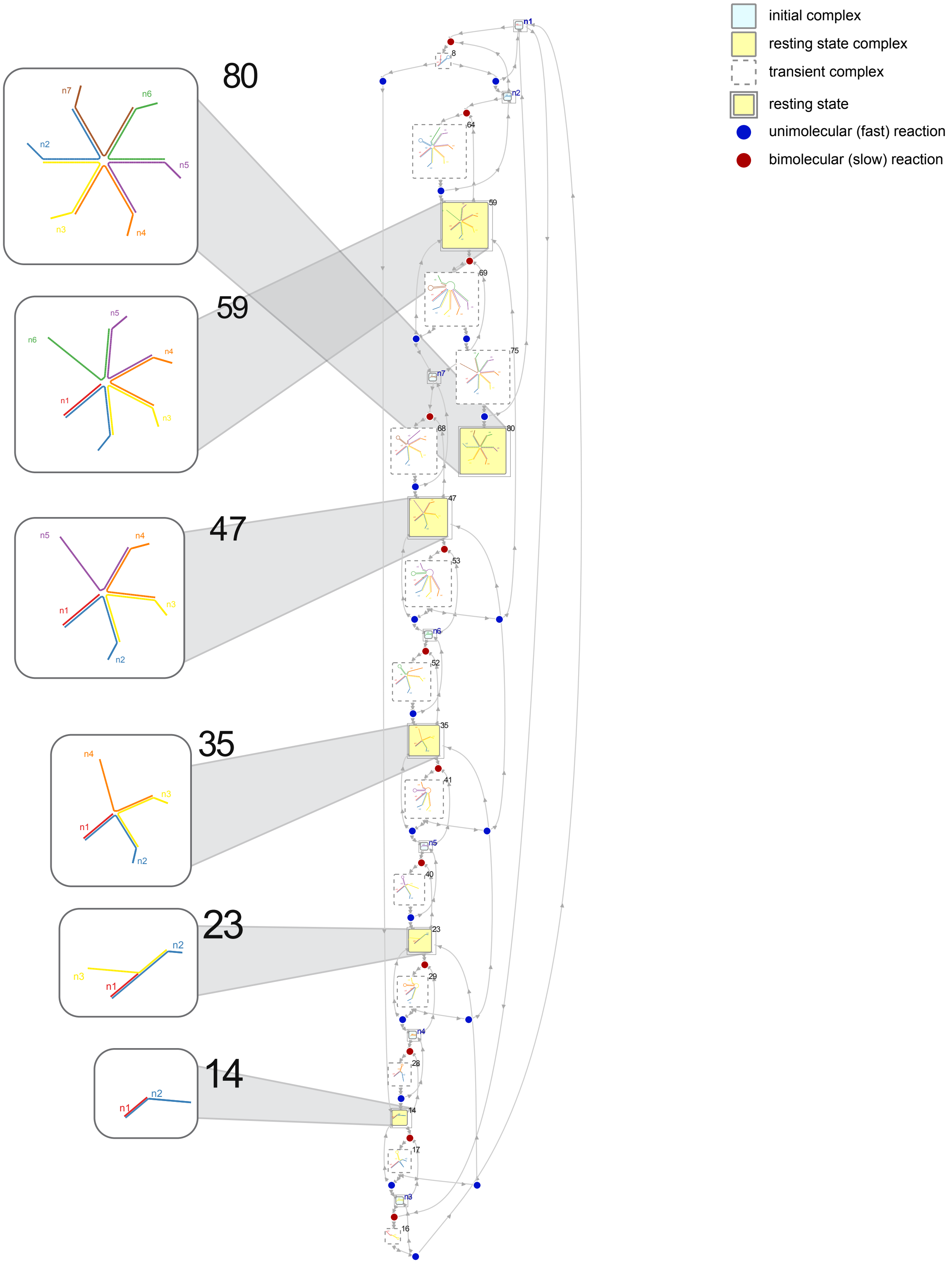

Figure S9: Full reaction enumeration for 6-arm junction. Important intermediates shown in detail on the left. (High resolution image—zoom in for detail)

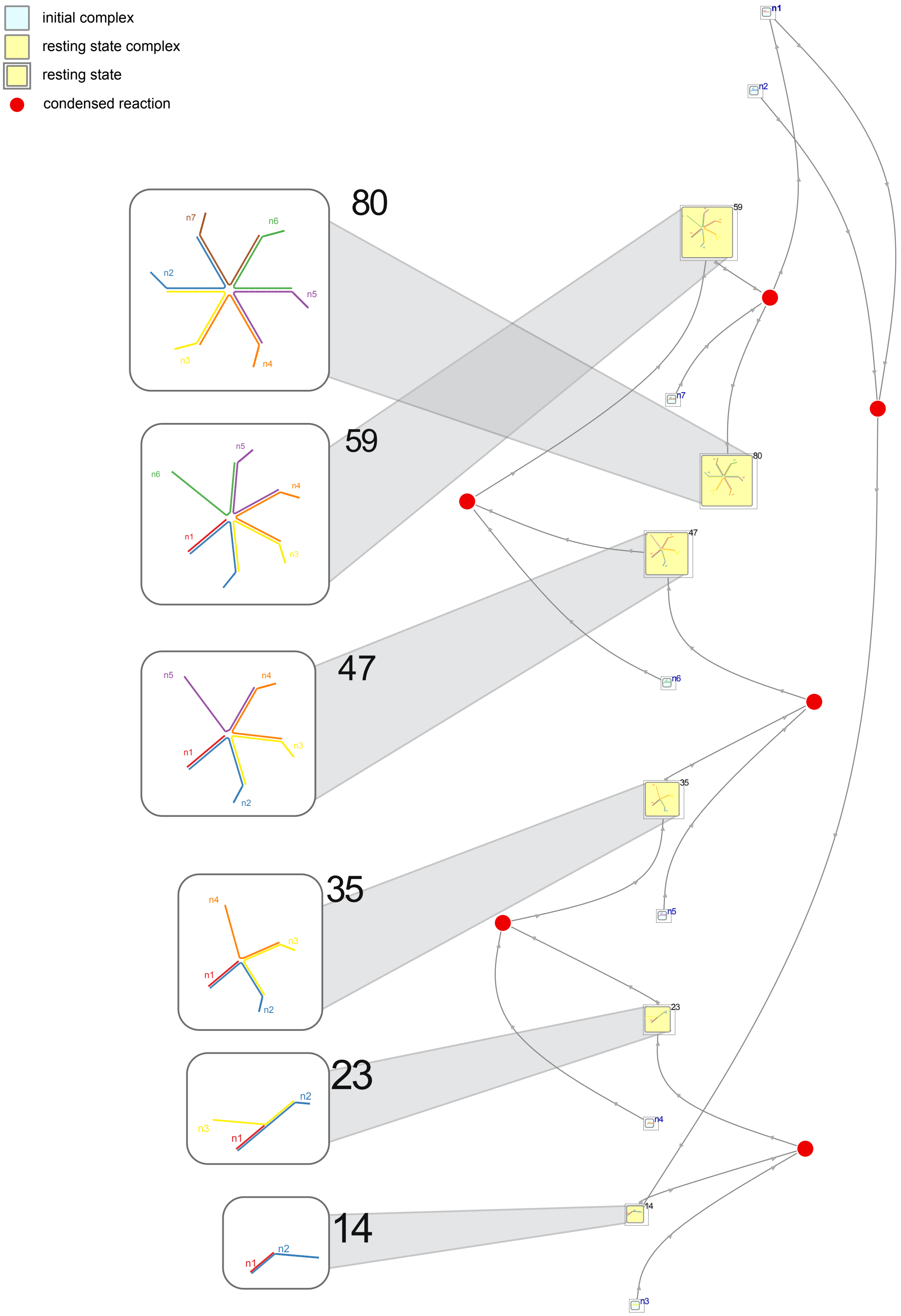

Figure S10: Condensed reaction enumeration for 6-arm junction. Important intermediates shown in detail on the left. (High resolution image—zoom in for detail)

use the stochastic sequence designer DD (see Sec. 4.3) to produce a set of sequences. These sequences are shown in Fig. S11. We could additionally have used the NUPACK thermodynamic sequence designer [18], or the Multisubjective multi-paradigm sequence designer [3]; for the sake of simplicity and speed, we will use DD for these demonstrations. In each demonstration, we have allowed DD to mutate the system until 1000 consecutive mutation attempts failed to improve the heuristic score for the system[2]. For the sake of demonstration in the following paragraphs, no additional attempts were made to manually tune the sequences further (e.g. to reduce undesired secondary structure or to eliminate long runs of identical nucleotides).

Before these sequences are synthesized and evaluated in a laboratory, we would like to perform some sort of evaluation of their suitability. One option is to attempt a base-resolution kinetic simulation. For instance, Schaeffer's "Multistrand" simulator [19] uses a base-resolution kinetic and thermodynamic model to simulate multiple trajectories through the state space of possible secondary structures. By aggregating many such trajectories, the simulator can produce a statistically-correct sampling of the underlying Markov process. The use of such a simulation for evaluation is theoretically important, but it is computationally expensive for even small systems. For this paper, we will satisfy ourselves with a more heuristic approach to sequence evaluation.

Heuristically, we can evaluate the sequences designed by DD by examining their thermodynamic properties; to do this we will use the NUPACK web server [2]. NUPACK utilizes an efficient algorithm to compute the thermodynamic "partition function" and the minimum free energy (MFE) secondary structure for each complex, across the full ensemble of possible secondary structures [20, 21]. The partition function calculation allows NUPACK to predict the expected concentrations of each possible complex (up to some specified maximum size) in a test tube at equilibrium. Additionally, NUPACK can calculate the probability with which each possible base pair is formed in a given structure [22]. Together, this information allows us to evaluate how "well-designed" a set of sequences are—how well their thermodynamically-predicted structure corresponds to their intended structures.

A rigorous metric for this would be the "normalized ensemble defect"—"the average number of incorrectly paired nucleotides at equilibrium evaluated over the ensemble of unpseudoknotted secondary structures" [18]. The ensemble defect is most useful when the intended structure is relatively small, and the intended structure is the thermodynamic minimum free energy configuration for the system. For many dynamic systems, this is not a useful metric (since the interesting behavior of the structure happens in the non-equilibrium dynamics—this is the case, for instance, with the exponential amplifier described in Sec. S2.3 below). For other systems (including this one), the metric is not practical to evaluate for the target structure itself, as it takes $\Theta(N^3)$ time to compute the ensemble defect for a system of N total nucleotides. The time to compute the partition function for the six-arm junction system (allowing complexes up to seven strands to form), using the NUPACK cluster, is on the order of days. Systems such as the tetrahedron are intractable (both due to the cubic complexity of the ensemble defect evaluation and the difficulty handling pseudoknots).

Even if thermodynamic evaluation of the target structure (the formed six-arm junction) is infeasible, we can at least evaluate the design of the *monomer* structures; this is the analysis presented in Fig. S12. Each panel shows (on the left) the minimum free energy structure of the indicated strand, and (on the right) the pair probabilities for each possible nucleotide pair. On both sides, bases are colored according to their probability (with dark red being high probability and dark blue being low probability).

We see immediately a few problems; for instance, the MFE structure of the single-stranded initiator `n1` is not linear—there are three bases of high probability secondary structure. Secondary structure in toehold regions is problematic, as it can slow the kinetics of toehold-mediated branch migration considerably by reducing the effective toehold length [23]. Examining the sequence, we see that there is a sequence of three consecutive A's bound to three consecutive T's. DD attempts to eliminate these types of motifs (repeats of multiple nucleotides), but sometimes it is difficult.

We might also be concerned that, in `n2`, `n4`, and `n5`, the first base pair of the hairpin stem (the first nucleotide on the 5' end) is not formed in the MFE structure. This makes it more likely that the hairpin stem will spontaneously "breathe" due to thermal motion and expose one of the toehold sequences (which should be protected by the duplex). This can cause "leakage"—activation of downstream components of the system before their upstream activators are present. Leakage reduces the "triggered" nature of the system, and increases the background level of structure that is formed absent any initiator. Leakage has been a

[2]See Sec. 4.3 for a discussion of what this score includes, or Zhang's original whitepaper [1] for more details on how it is calculated.

persistent problem for systems like this one that based on the hybridization chain reaction concept [4,24,25]. The introduction of the short, 2 nucleotide “clamp” regions (which are independent of any toehold sequences) is one attempt to combat this problem, but more careful sequence design is required for this approach to work.

The examination of these types of issues would likely drive a molecular programmer to either return to DD and further mutate the sequences for the system, or to manually tweak the sequences and perform another analysis with NUPACK. DyNAMiC Workbench facilitates both approaches—sequence design in DD can be paused, saved, and resumed at any time, and sequences can be manually edited (including locking bases against mutation by DD). DyNAMiC Workbench also makes it easy to submit sequences to the NUPACK, Mfold, and RNAfold web servers for analysis [2,26,27]. Another option would be to consider using Multisubjective or the NUPACK designer for sequence design. DyNAMiC Workbench makes it easy to submit the same segment-tier design to multiple sequence designers; we could then perform an analysis on each and compare the results.

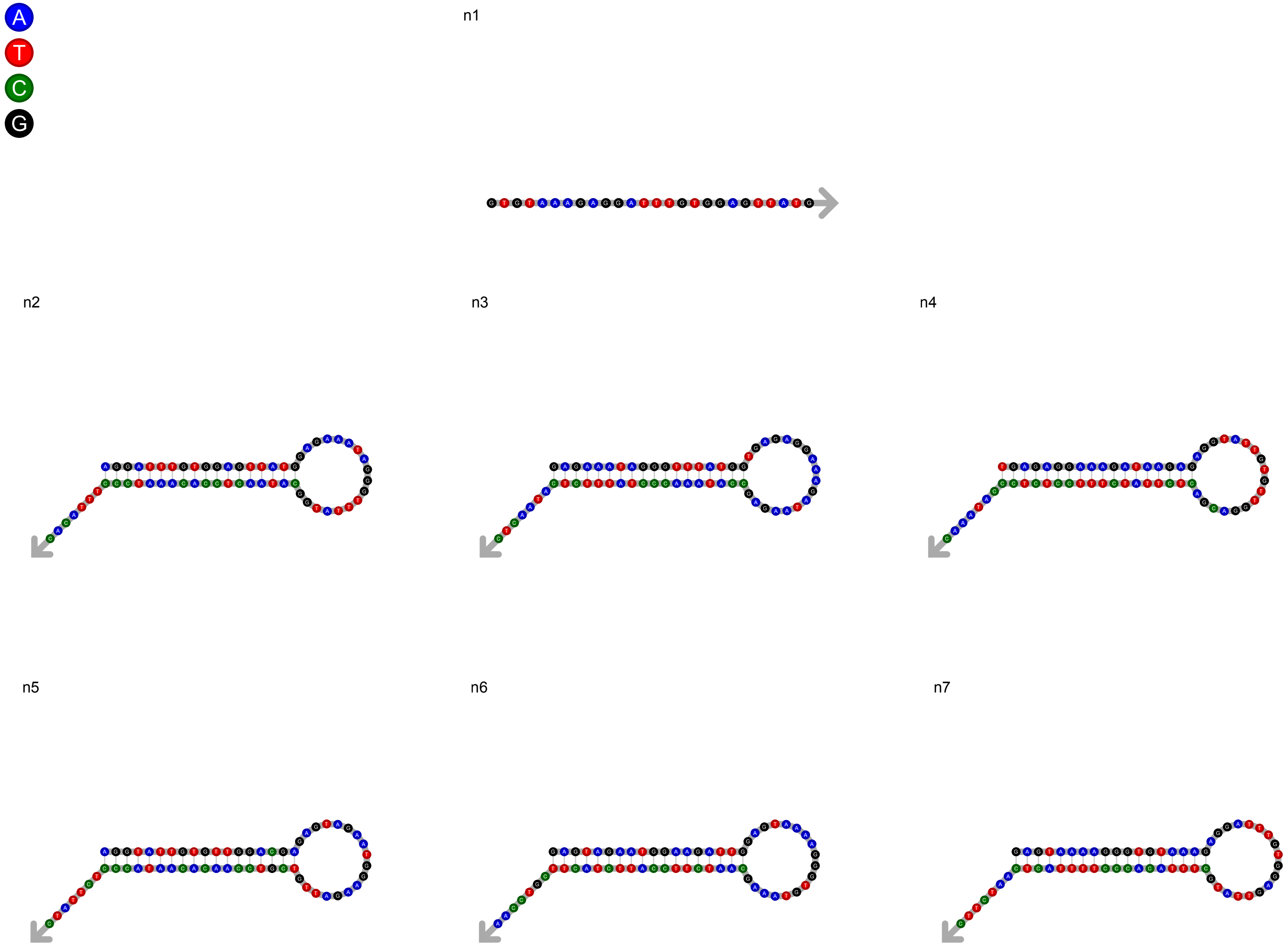

Figure S11: Sequences for 3-arm junction, as designed using DD, terminating after 1000 interations with un-changed sequences.

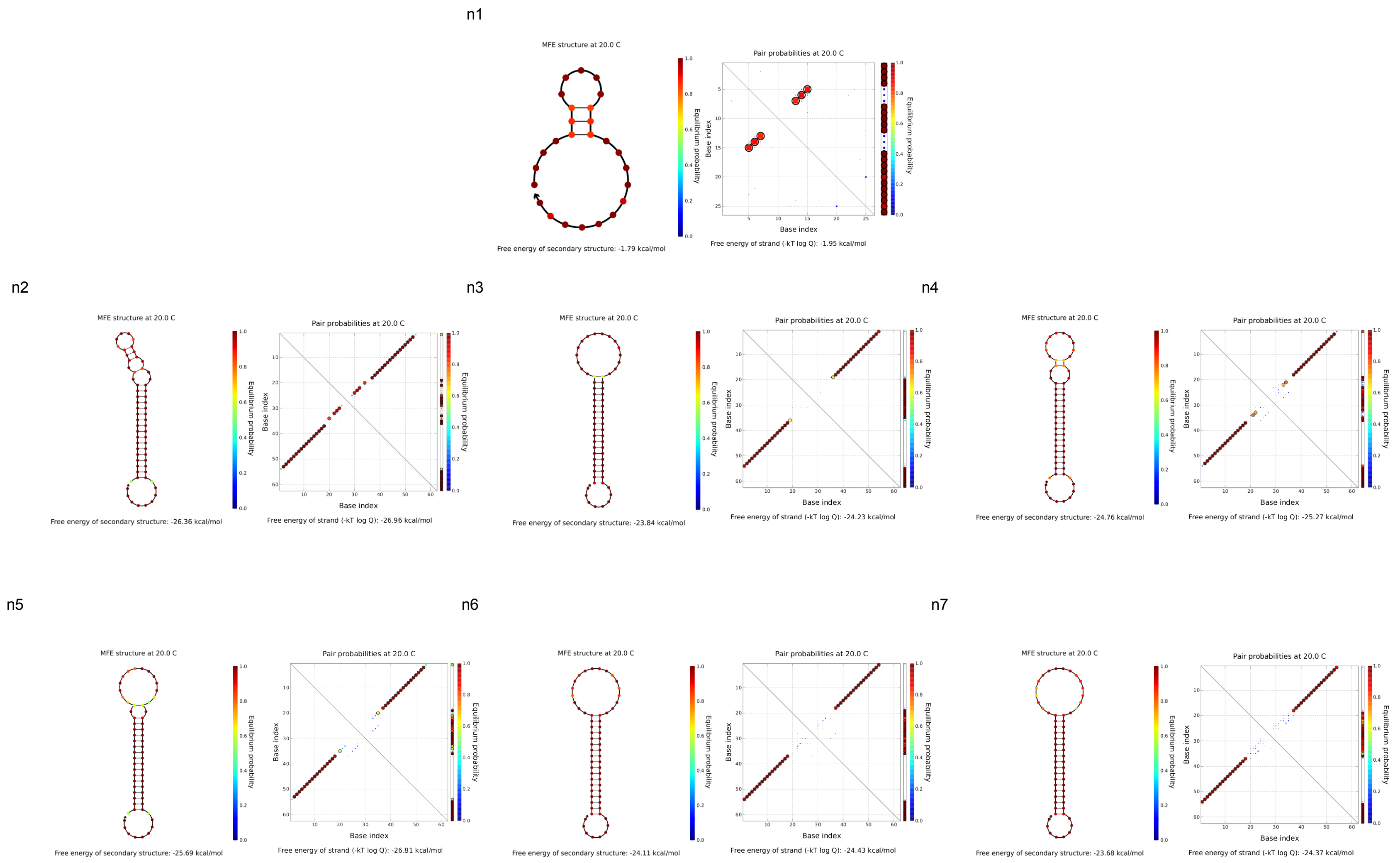

Figure S12: Thermodynamic analysis of DD-designed sequences (Fig. S11) for 6-arm junction, performed using the NUPACK web server [2]. For each strand, the minimum free energy (MFE) structure is shown on the left, while the probability of each possible base pair is shown in the plot to the right. Probability is indicated by color, with high probabilty bases shown in deep red and low probability bases shown in dark blue.

S2.2 Bounded Dendrimer

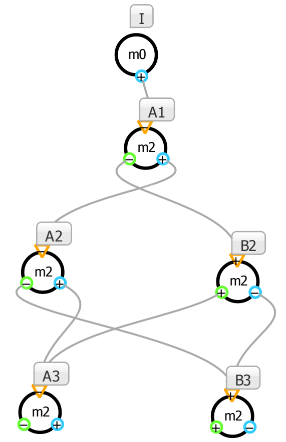

Figure S13: Nodal representation of bounded dendrimer

Next we consider the construction of a binary molecular tree of a prescribed size. Like junction structures, branching "dendrimers" have attracted wide interest as fundamental structural units for nanotechnology [28]. Additionally, there has been some recent interest in the use of dendritic structures in a clinical diagnostic setting for highly-specific detection of nucleic acid signals [29]. This application relies on the exponential amplification potential of such structures; a G-generation dendrimer will contain $2G-1$ distinct species, but can form a binary tree containing 2^{G-1} strands. Previous work constructed these structures by either incorporating chemically- modified branchpoint nucleosides [30, 31] or employing serial ligation steps [32]. However, Yin et al. showed that a programmed assembly pathway could be developed that formed a dendritic structure upon addition of an initiator, using only unmodified nucleosides [4]. Their design operates on the same principle of triggered assembly as the three- and six-arm junctions—hairpins serve as meta-stable monomeric species that remain stable until the addition of a single-stranded initiator sequence. Yin et al. experimentally demonstrated a $G = 4$ dendrimer; we will follow the their approach, but for simplicity of analysis we will construct only a $G = 3$ dendrimer.

The Nodal design for the dendrimer (Fig. S13) again begins with an initiator (`I`); the initiator opens a two-output node `A1`[3], which serves as the initial branch-point. In each stage, or "generation" of the dendrimer, there are two additional nodal species—each of which is connected to *one* of the outputs on *each* node in the previous generation. For $G = 1$, this means that `A2` and `B2` are each connected to one of the two output ports of `A1`; for $G \geq 2$, each node is joined to *both* growing arms of the dendrimer—that is, `A3` is connected to both `A2` and `B2` (likewise for `B3`). This pattern allows formation of the exponentially-branching pattern.

This system uses the two-output node type `m2`; this node type is implemented by a single hairpin with an elongated "tail" region, which serves as an additional output domain. Importantly, the toehold for this tail is sequestered *inside* the duplex stem, meaning that the entire domain is oriented opposite the other output domain (the toehold is closer to the negative pole than the positive pole). We have colored the port corresponding to the loop domain blue, and the port representing the tail domain green.

Again, a segment-tier representation of the entire system is produced by the Nodal compiler (Fig. S14). Notice the problem of propagated sequence complementarities is again apparent in this system—`5` and `6` appear in the $G = 3$ nodes (`A3` and `B3`), while their complements `6*` and `5*` are single-stranded in `A1`. However, note that this will again only affect the kinetics of the system (since these are short, toehold-length

[3]For this system, we will name the nodes following the convention in [4], rather than our usual nx, to emphasize the generation to which each node belongs; therefore `A1` is a $G = 1$ node, `A2` is $G = 2$, etc.

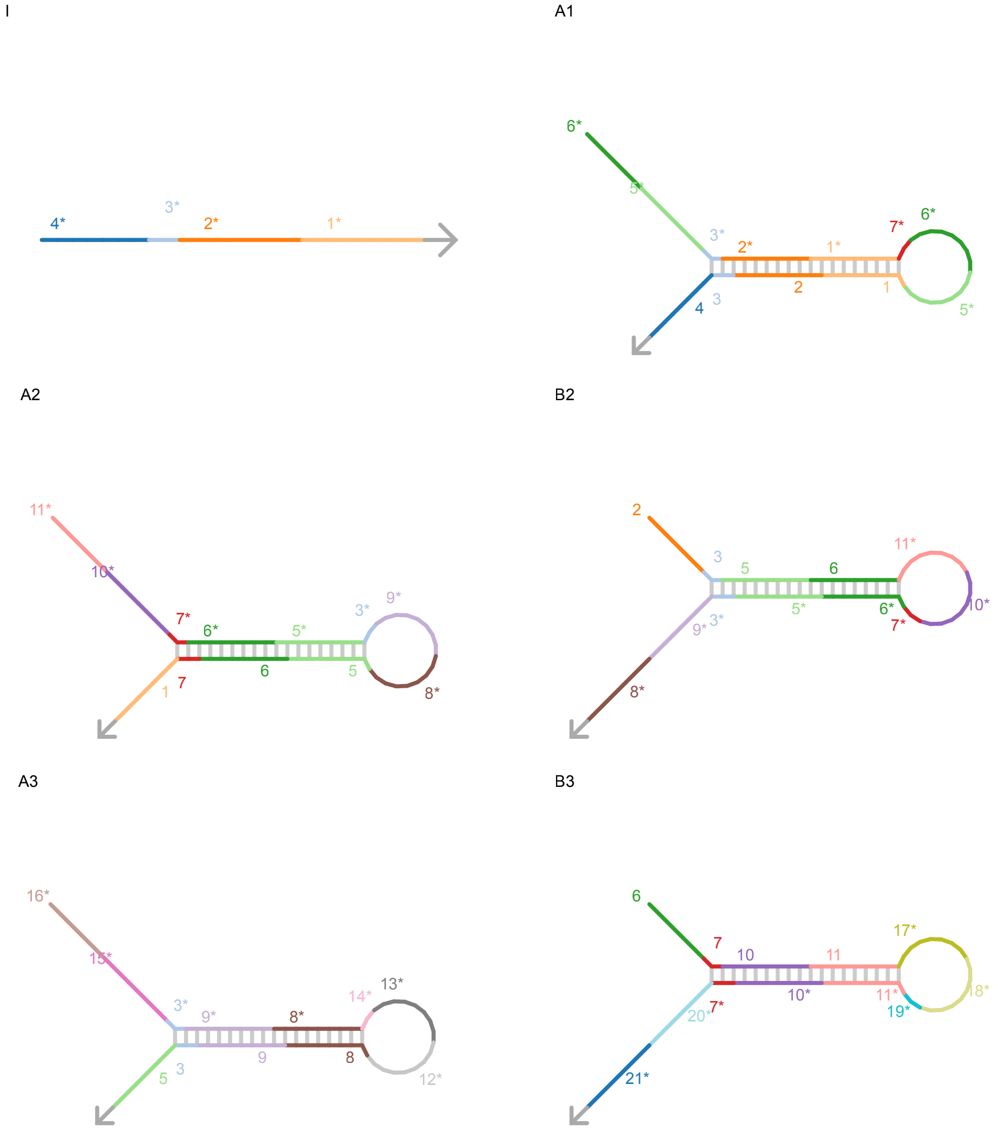

Figure S14: Segment-tier representation of bounded dendrimer

segments, and neither strand has an adjacent, longer recognition segment (or series of recognition segments) that can be displaced by branch migration).

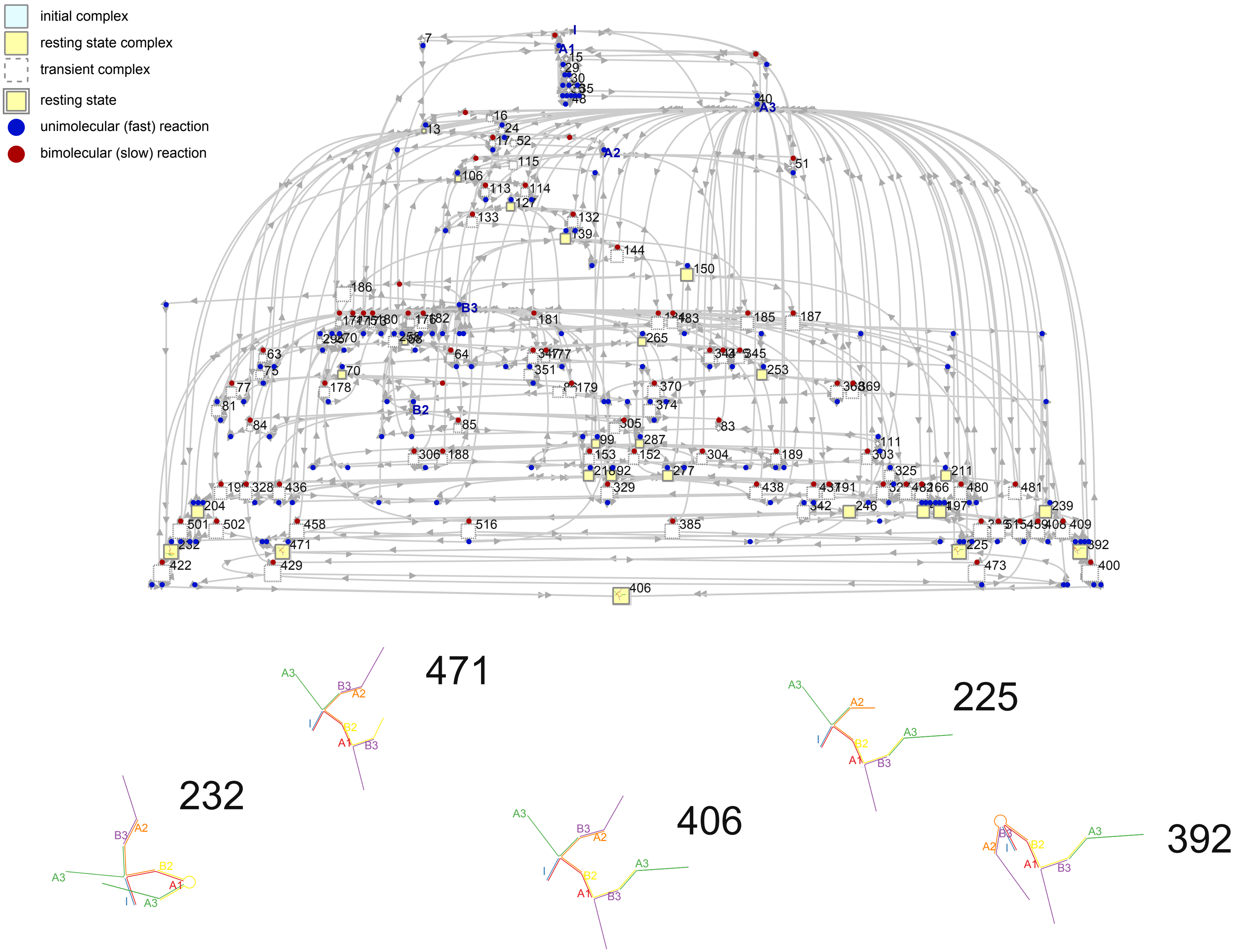

Figure S15: Full reaction enumeration for bounded dendrimer (High resolution image—zoom in for detail)

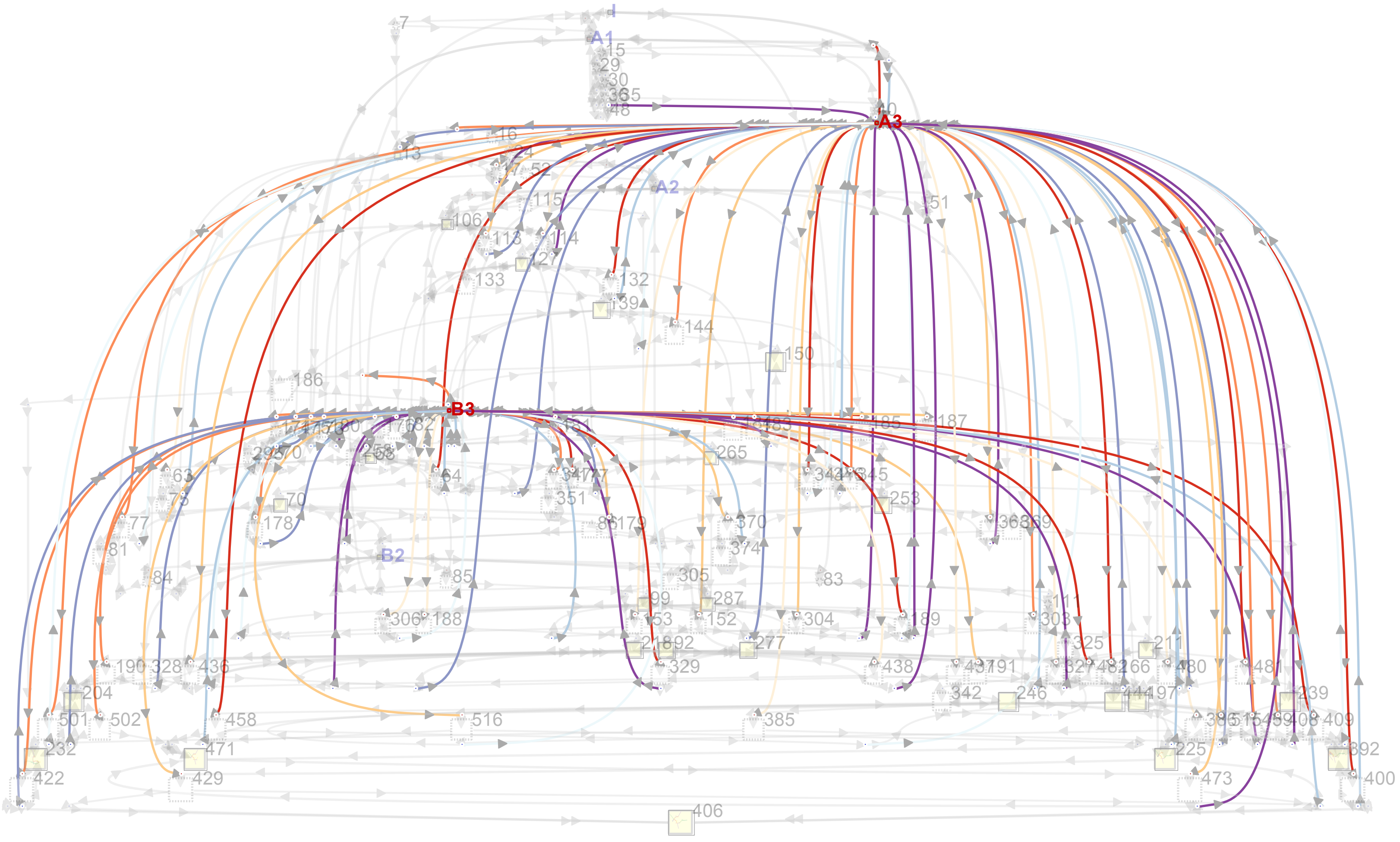

Figure S16: Full reaction graph for bounded dendrimer, highlighting monomers A3 and B3. (High resolution image—zoom in for detail)

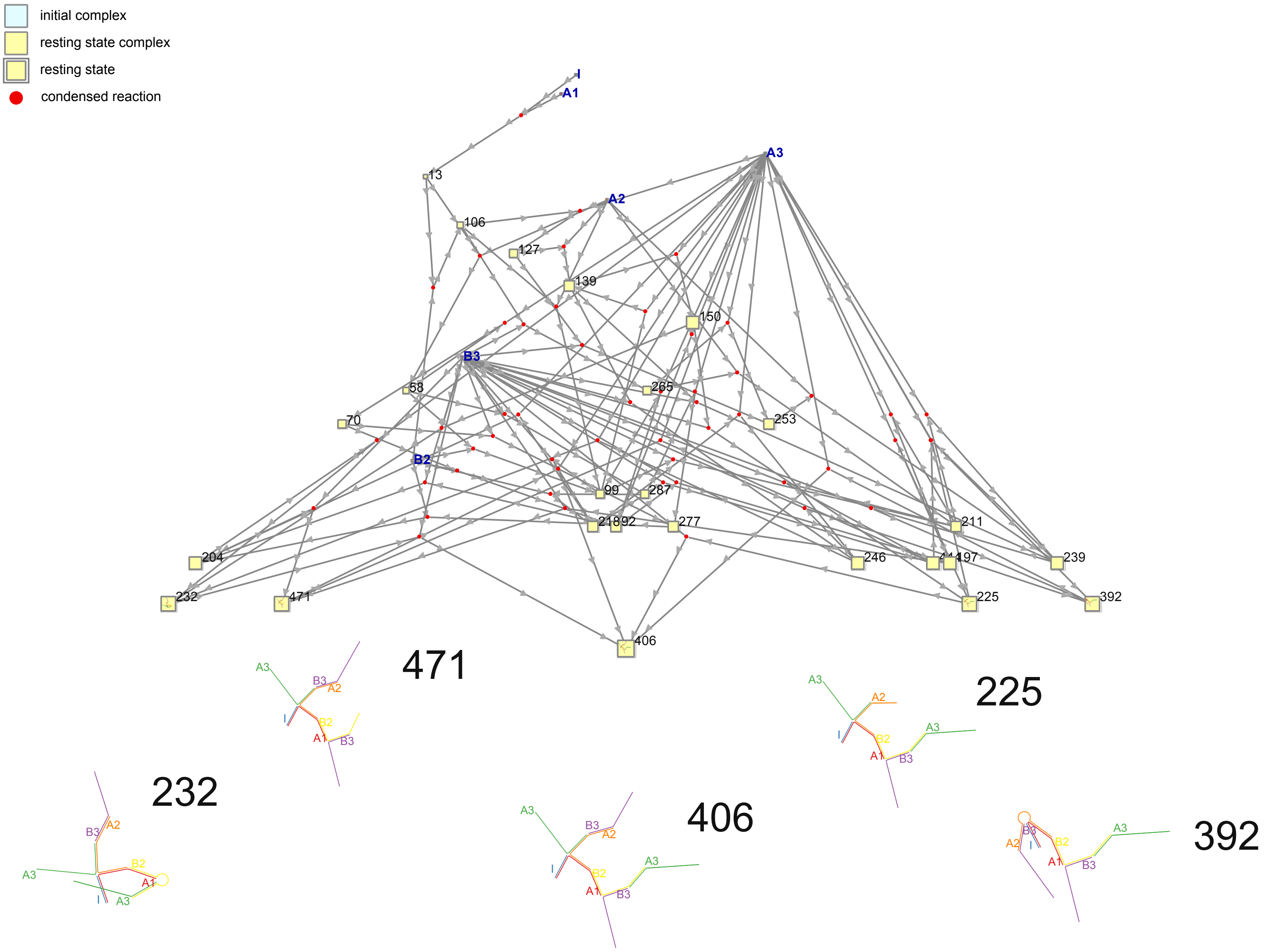

Figure S17: Condensed reaction enumeration for bounded dendrimer (High resolution image—zoom in for detail)

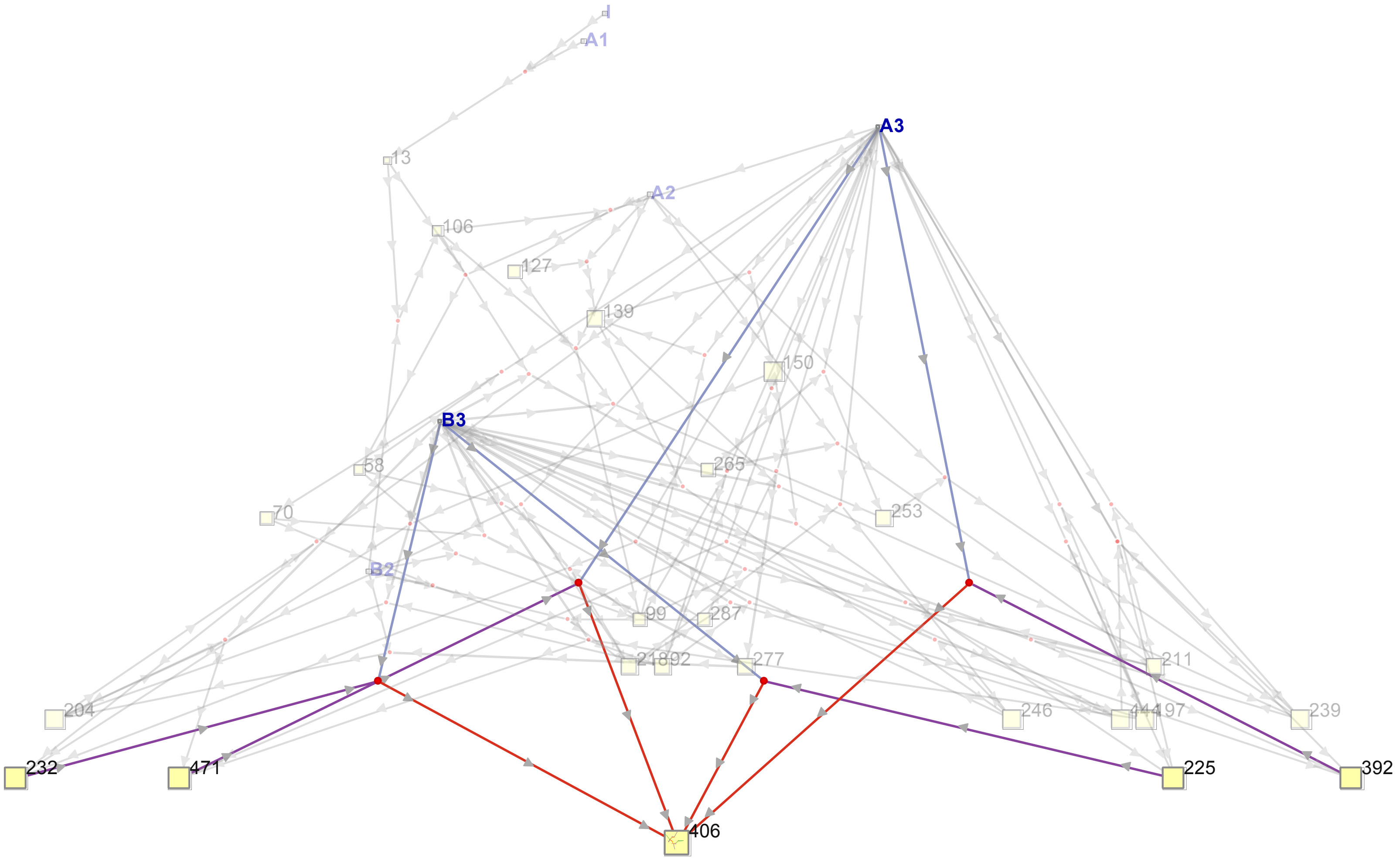

Figure S18: Condensed reaction enumeration for bounded dendrimer highlighting paths to final product (High resolution image—zoom in for detail)

The details of the full reaction graph (Fig. S15) for this system are very difficult to interpret without the interactive utilities provided by DyNAMiC Workbench; we notice, however, that all paths converge to a single target product (complex `406`) at the bottom of the graph; upon inspection, we see that this is in fact our target product—the $G = 2$ dendrimer. In DyNAMiC Workbench, we can highlight the reactions producing and consuming a particular complex—reactions *producing* the indicated species are connected with cool-colored (blue, purple, etc.) lines, while reactions *consuming* the species are warm-colored (red, orange, etc.); we can see that the third-generation species `A3` and `B3` are the most highly-connected components of the graph (Fig. S16). This is to be expected, since the latest-generation species should participate in the greatest number of different reactions (interacting with each possible branch of the $G = 2$ tree).

Examining the condensed reactions (Fig. S17) reveals there are four parallel pathways to the final product, via four distinct intermediate products: `232`, `471`, `225`, `392`. Inspecting these complexes, we see they each correspond to the final structure, but are each missing either `A3` or `B3` on one of the arms (Fig. S18). We can similarly examine previous generations of the dendrimer in the condensed view.

Finally, we use DD to produce a set of sequences to implement this structure (Fig. S19). Again, there are issues with the sequence design revealed during analysis (Fig. S20). In particular, we note the appearance of secondary structure in the toehold segment `2` of `B2`. The appearance of low-probability secondary structure in the "tail" regions (e.g. on the 3' end of `B3`) will likely not significantly affect the kinetics of branch migration [23].

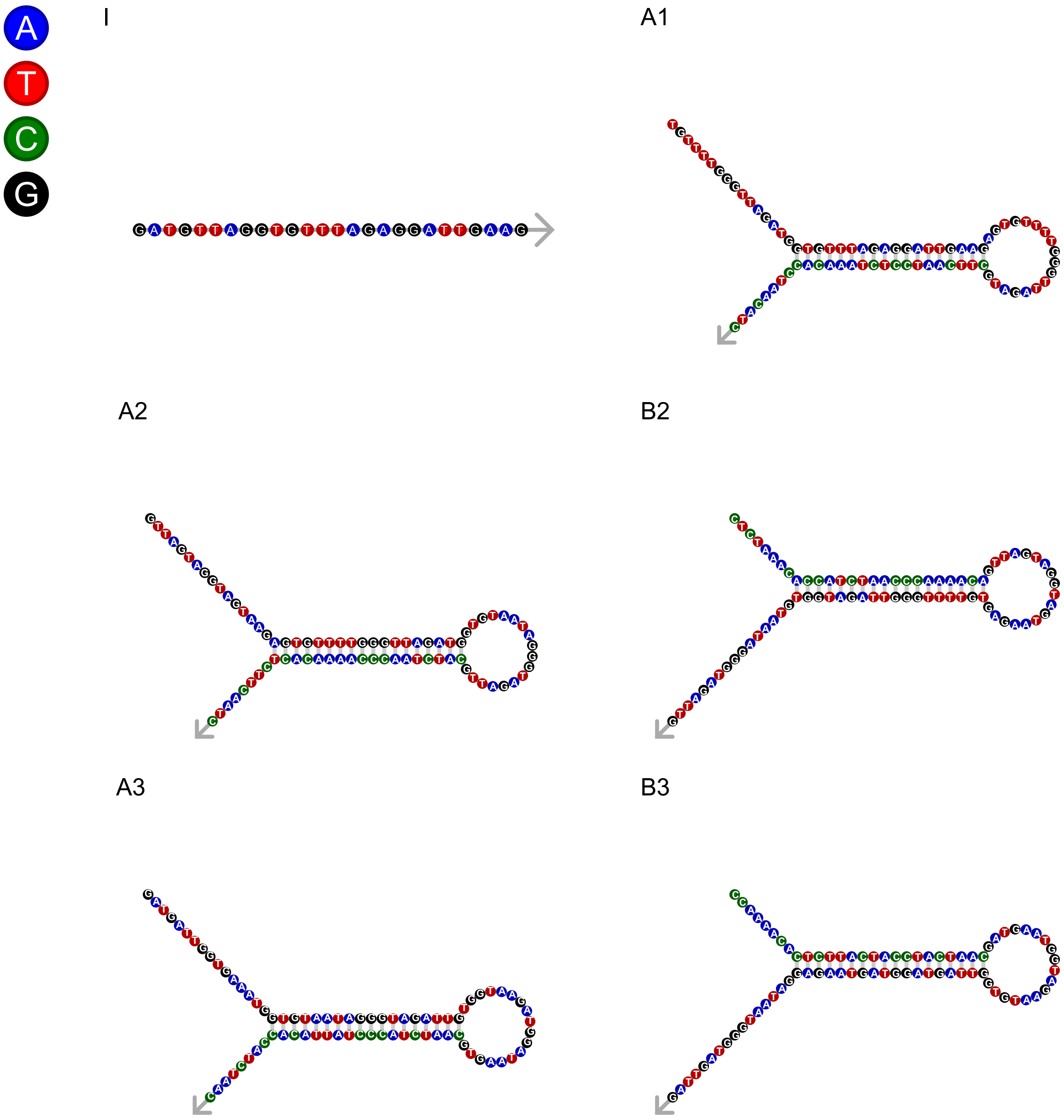

Figure S19: Sequence-tier representation of bounded dendrimer

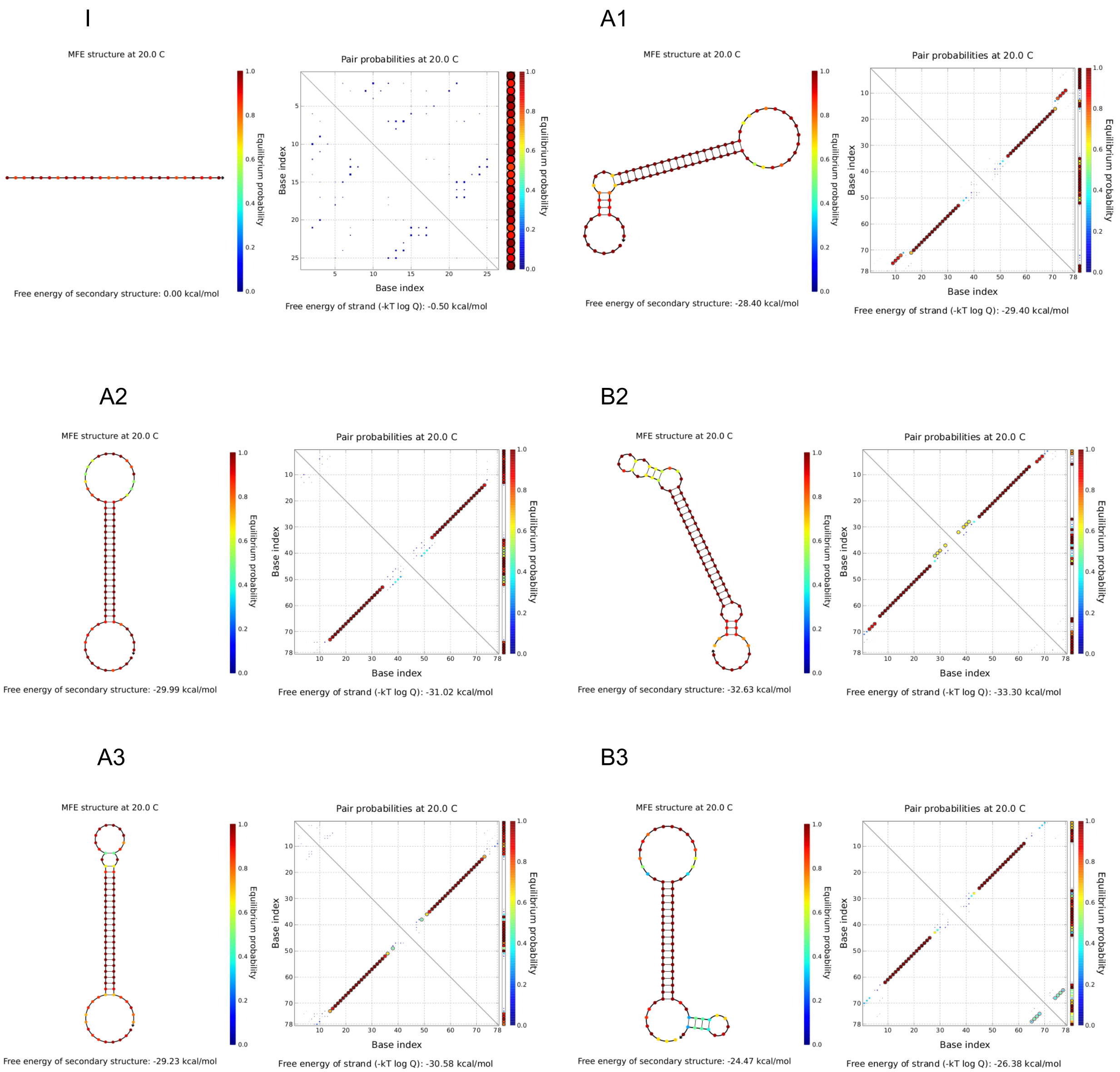

Figure S20: Thermodynamic analysis of DD-designed sequences for the bounded dendrimer

S2.3 Catalytic Amplifier

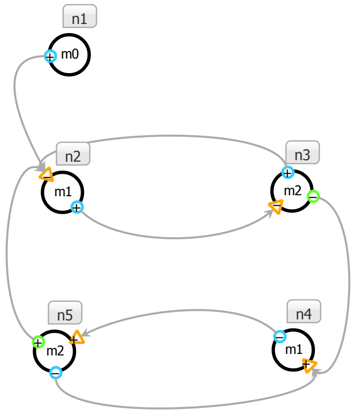

Figure S21: Nodal representation of catalytic amplifier

For the previous structure, we considered an exponential amplification that relied on growth of an exponentially-large structure; exponential growth is not a requirement for amplification. Here we demonstrate a system which performs exponential amplification via cross-catalytic self-assembly. By this, we mean that the system forms two primary products (complex `30` = `n2` + `n3` and complex `75` = `n4` + `n5`), each of which *catalyze the formation of one another.* This means certain branch migration events cause dissociation of previously-assembled complexes, avoiding the requirement to build an exponentially large structure.

The nodal representation demonstrates this behavior (Fig. S21). `n1` opens `n2`, which begins the cycle. `n2-n3` form an irreversible bond, and `n3` displaces `n1`. `n3` catalyzes opening of `n4`, but is displaced by `n5`. `n4-n5` form an irreversible complex, which further triggers opening of `n2` (this is the "cross-catalysis").

A segment-tier representation is generated automatically by DyNAMiC Workbench (Fig. S22).

Both the full and condensed reaction networks capture the cyclic behavior of the system (Fig. S23, Fig. S24). Considering the condensed system, we can see that, once formed, complex `30` acts as a catalyst to form the other target complex, complex `75`: first `30` opens `n4` (forming `43`), then `43` binds `n5`, producing complex `75` and liberating the catalyst `30`. The opposite process occurs near the bottom of the network, with `75` opening `n2` to form the three-stranded intermediate `96`, then dissociating to liberate both target complexes (`30` and `75`). The four-stranded intermediates of this process can be seen in the full reaction view (`63`, `50`, `69`, `58`, etc.) A logical step to validate the exponential amplification behavior of this system would be to perform an ODE-based or stochastic simulation of the chemical reaction network produced by the enumerator—which can also estimate rates of the predicted reactions. The enumerator can produce a repreentation of this model using the Systems Biology Markup Language (SBML), which could be used to simulate the system using any available CRN simulation package. However, DyNAMiC Workbench has not yet integrated these facilities, so we presently omit this analysis.

We use DD to produce a set of sequences to implement this system (Fig. S25) and notice no major issues in a thermodynamic analysis of the sequences (Fig. S26). However, here we note that the lack of kinetic simulation tools integrated into DyNAMiC Workbench remains a major limitation—the real test of these sequences' performance as an exponential amplifier requires evaluation of the kinetics of the reactions. While the enumerator can estimate rates for these reactions (based only on the length of the segments involved), we would need a sequence-tier simulator such as Schaeffer's Multistrand to estimate the rates for reactions involving these particular sequences [19]. This type of analysis is an important area for future work.

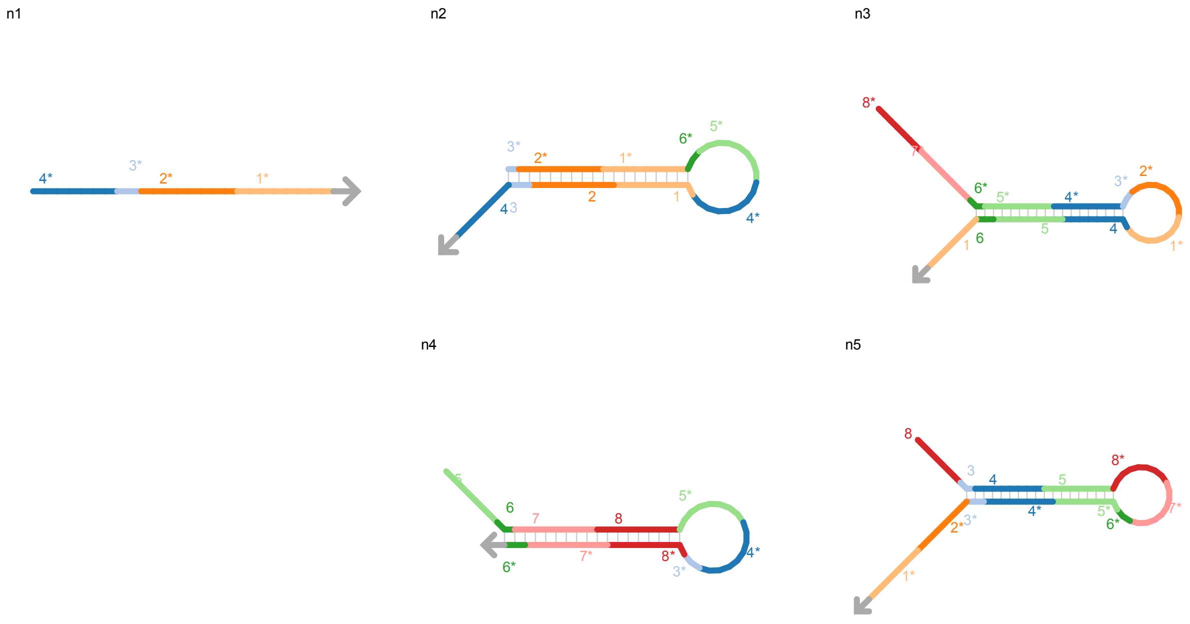

Figure S22: Segment-tier representation of catalytic amplifier

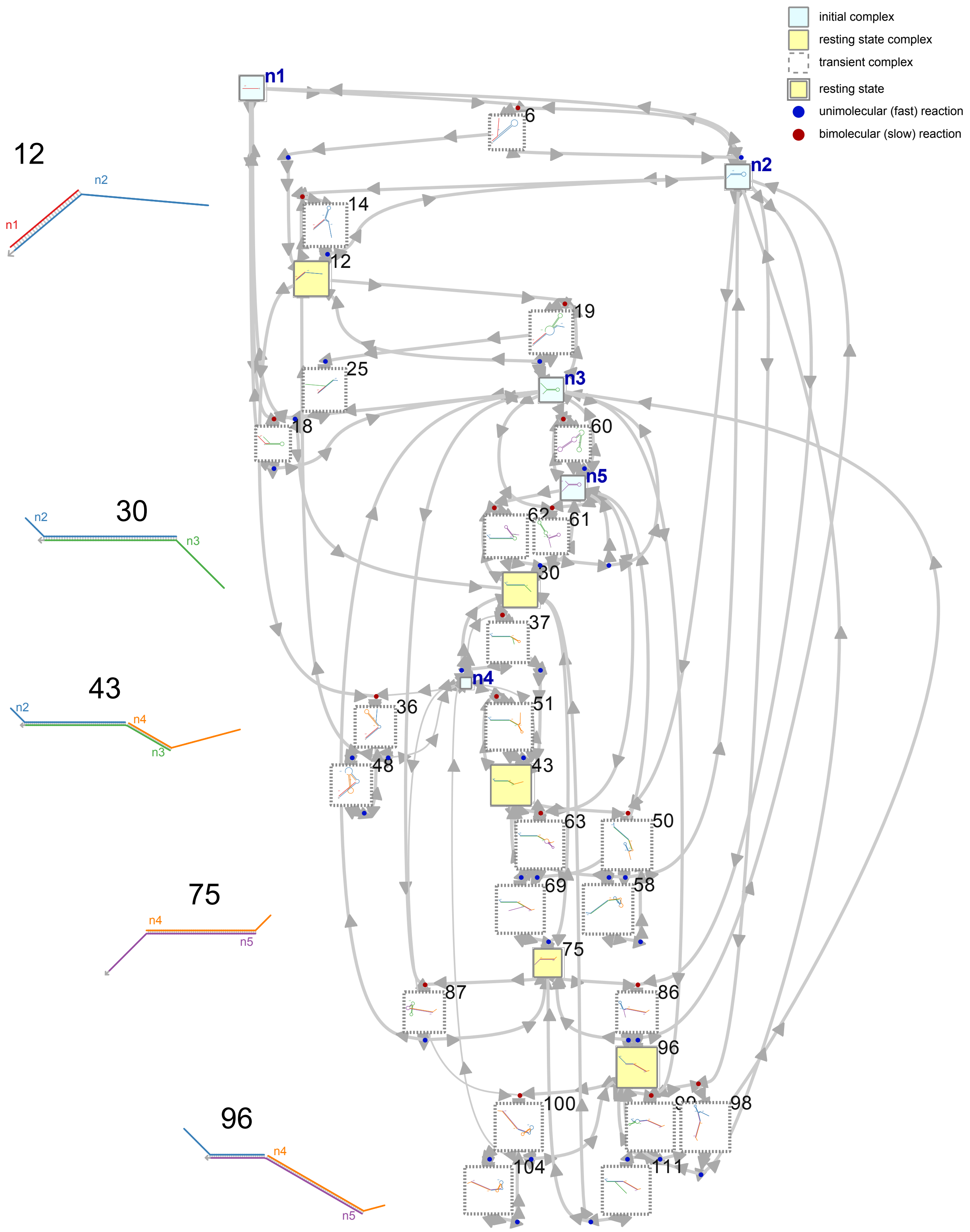

Figure S23: Full reaction enumeration for catalytic amplifier (High resolution image—zoom in for detail)

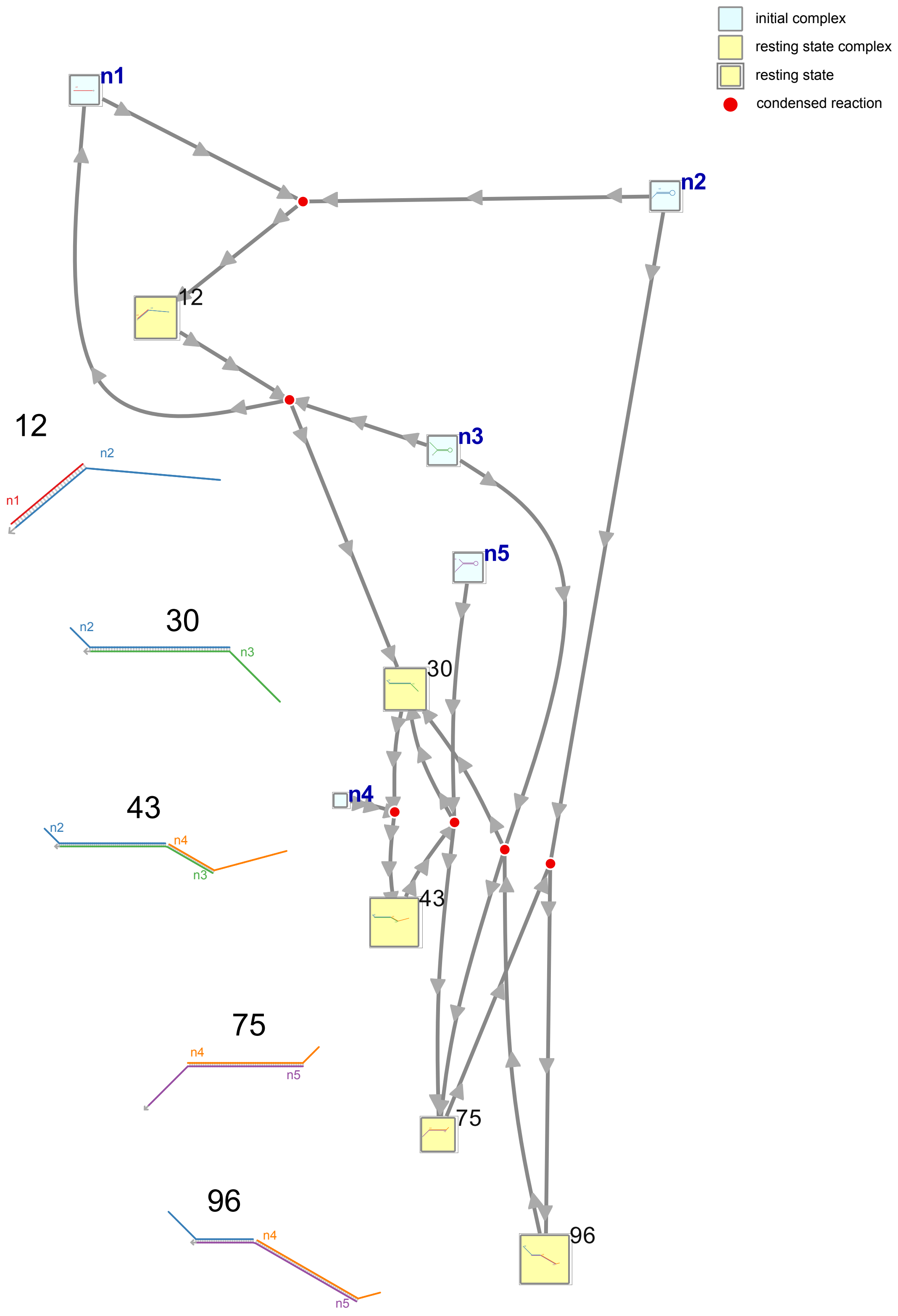

Figure S24: Condensed reaction enumeration for catalytic amplifier (High resolution image—zoom in for detail)

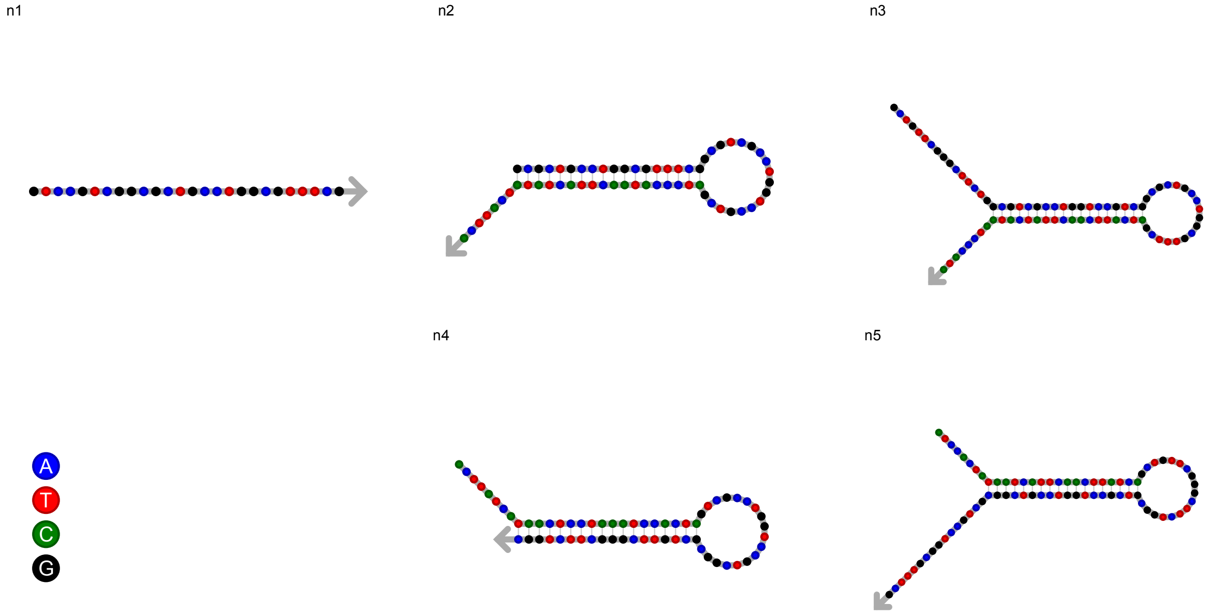

Figure S25: Sequence-tier representation of catalytic amplifier

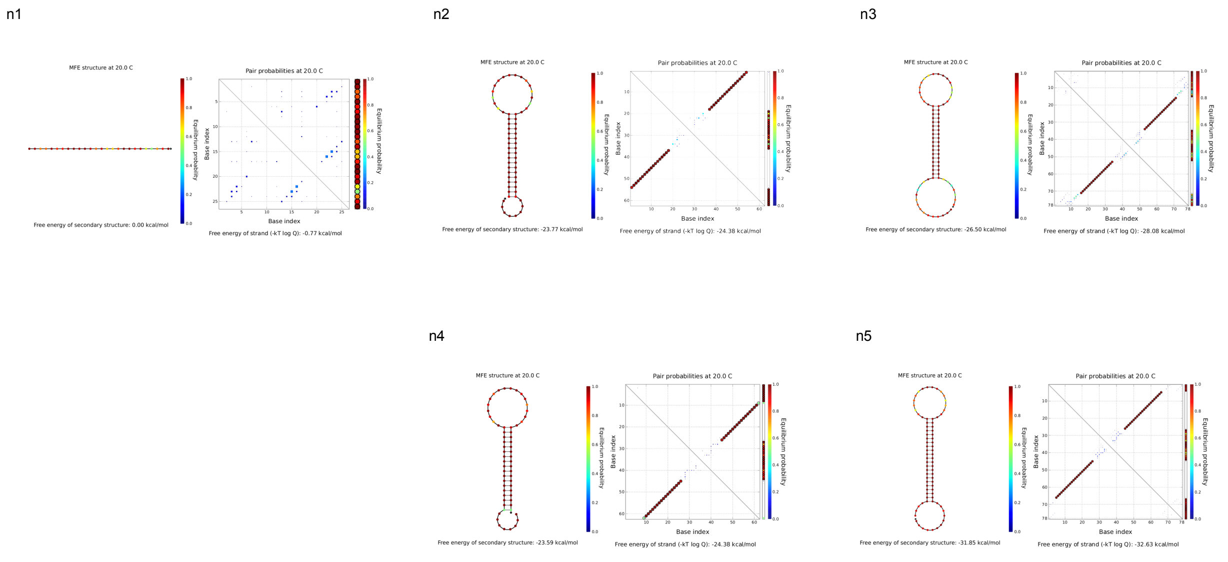

Figure S26: Thermodynamic analysis of DD-designed sequences for the catalytic amplifier

S2.4 Tetrahedron

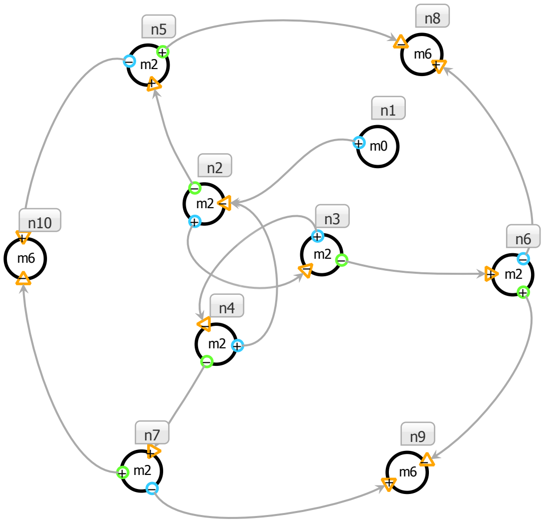

Figure S27: Nodal representation of tetrahedron

Finally, we will demonstrate the assembly of a more complex structure—a three-dimensional wireframe tetrahedron. While not approaching the size or structural complexity of shapes produced using thermodynamic methods such as DNA origami [33–36] or single-stranded tiles [37, 38], this structure importantly has a kinetically-controlled, prescribed assembly pathway. This structure was originally described in [5]; we present an implementation designed with DyNAMiC Workbench. Unfortunately, the final structure in this system is pseudoknotted (see Fig. S6d for an image of the intended 3D structure), meaning our enumerator cannot describe the entire assembly pathway. Instead, we provide a proof-of-concept implementation and demonstrate that key intermediate complexes are formed. Future work will be necessary to expand the facilities of the enumerator to handle pseudoknotted structures.

The nodal description of this system (Fig. S27) involves three "rings" of growth—the inner ring (`n2`–`n4`) begins with a program similar to the three-arm junction presented in the main text. We use the node type `m3` instead of `m2`, which gives each arm of the junction another output port to catalyze formation of the next ring (`n5`–`n7`). Finally, the two-input node type `m6` is used to link the three "arms" of the tetrahedron in a "ring-closing" reaction, forming the outer ring (`n8`–`n10`).

The node type `m6` is implemented as a duplex between a longer strand with two toeholds and a shorter "protector" strand. The protector strand cannot be displaced by binding of either of the individual toeholds alone—cooperative binding of *both* toeholds is necessary to displace the protector. This "cooperative hybridization complex" was first presented by [6], and serves as an important means for introducing synchronization into the assembly of the structure. The arms grow concurrently, but it is necessary for both terminal outputs to be present in order for the arms to be joined into a triangle—the cooperative complex enforces this sequence point. A segment-tier implementation of this nodal system was produced using DyNAMiC Workbench (Fig. S28).

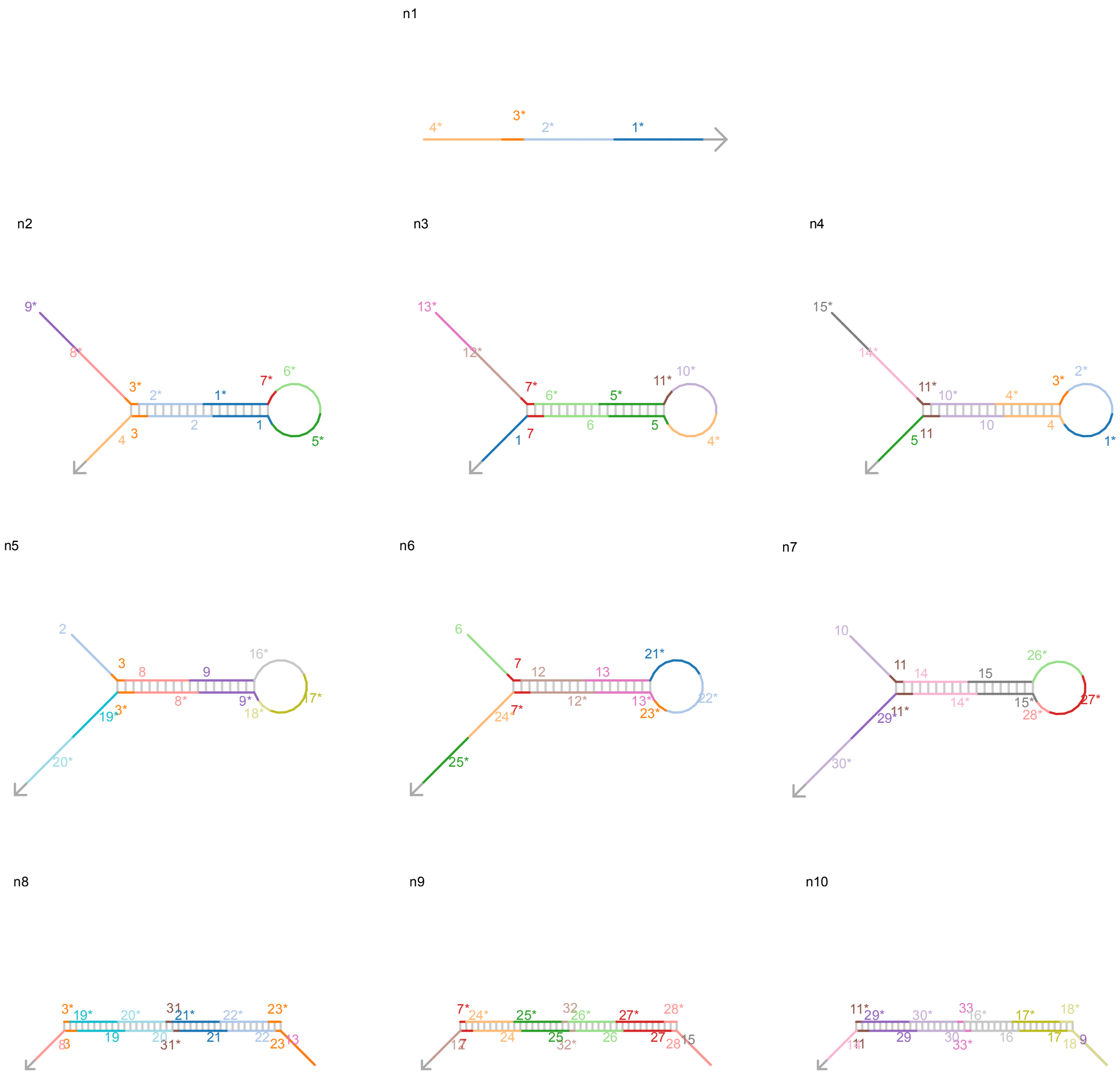

Figure S28: Segment-tier representation of tetrahedron

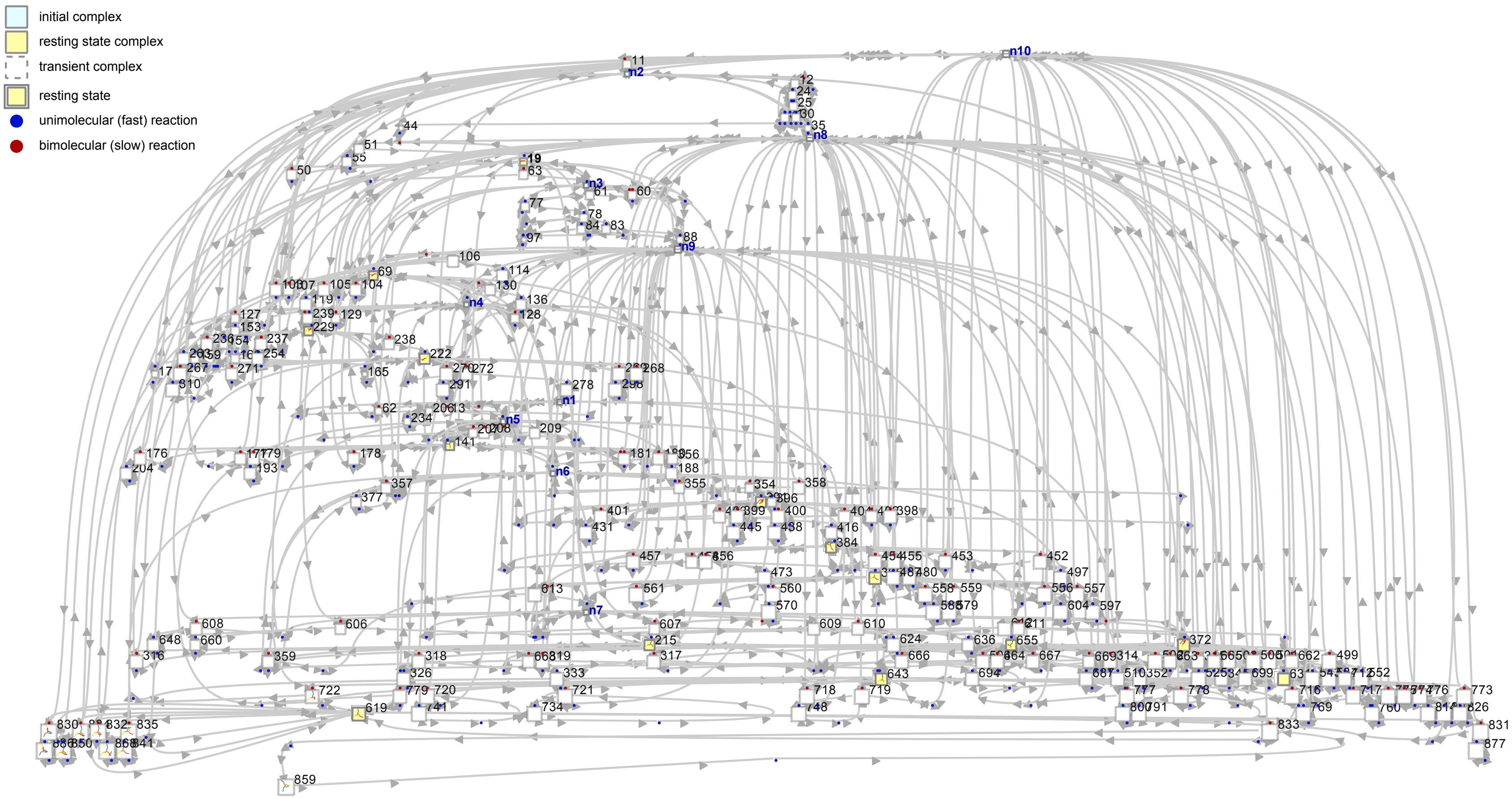

Figure S29: Full reaction enumeration for tetrahedron (High resolution image—zoom in for detail)

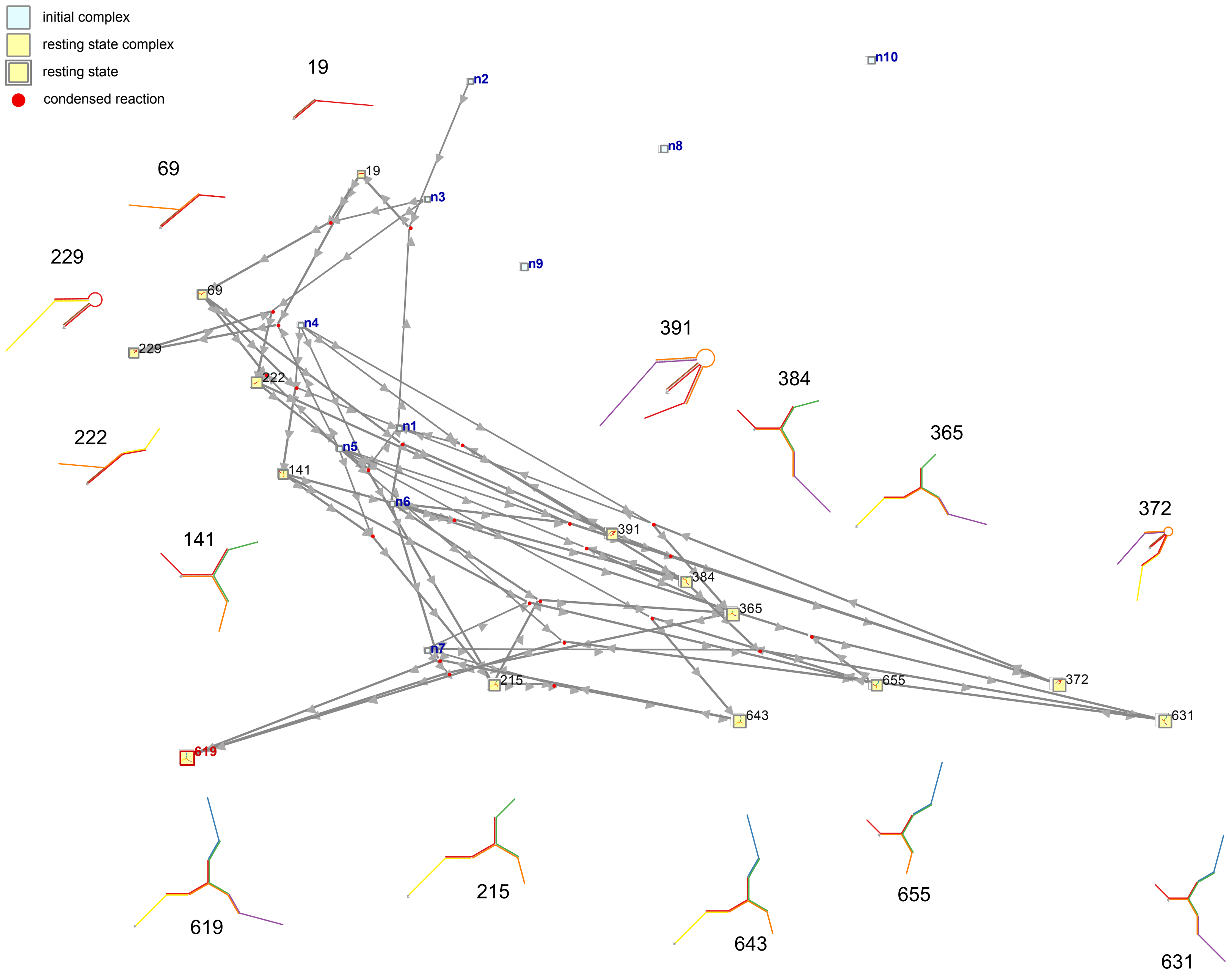

Figure S30: Condensed reaction enumeration for tetrahedron (High resolution image—zoom in for detail)

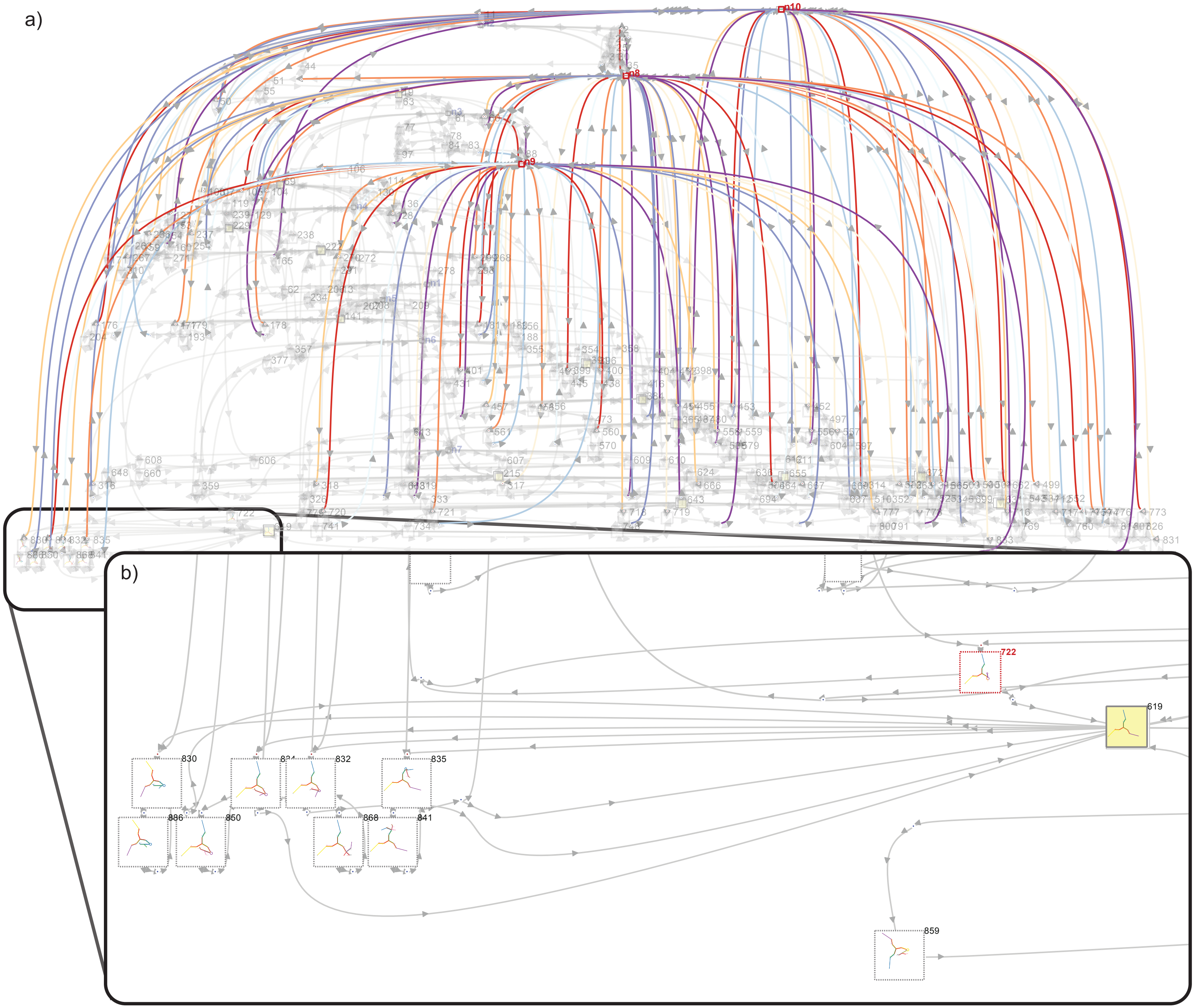

Figure S31: Full reaction enumeration for tetrahedron, highlighting reactions involving n8, n9, and n10 (High resolution image—zoom in for detail)

Much like the dendrimer, the full reaction graph for the tetrahedron (Fig. S29) is difficult to interpret; we will begin by considering the condensed reactions. In the condensed reaction graph (Fig. S30), we first notice that there are three orphaned complexes (top left) which do not participate in any condensed reactions. This means that these complexes either do not react, or they react only to form transient complexes which dissociate into their substituents. We realize that these orphaned complexes correspond to the cooperative hybridization complexes, `n8`–`n10`. This makes sense, given the discussed limitations of the enumerator—for these complexes to irreversibly associate, *both* of their toeholds would need to bind—forming a pseudoknotted triangular structure. We see that the terminal resting state complex, shown in the lower-left, is comprised of the first two rings of the tetrahedron—`n2`–`n7`, but does not include strands from any of these cooperative complexes.

Examining the full reaction graph, and highlighting the reactions in which `n8`, `n9`, and `n10` participate, we see that they interact transiently with many intermediates (Fig. S31a)—corresponding to the binding of one of the two toeholds on the cooperative complex to an immature tetrahedron. Zooming in on the lower left corner of this graph (Fig. S31b), we can observe several of these intermediate structures (`830`, `834`, `832`, etc.). Each of these structures has, at most, one of the cooperative complex strands bound.

We use DD to produce a set of sequences implementing the structure (Fig. S30), and again observe several issues with toehold secondary structure—in particular nodes `n2`, `n3`, `n4`, and `n6`, which have base pairs in the toeholds with equilibrium probabilities of greater than 50% (Fig. S33). The Multisubjective designer was originally developed to handle exactly these types of issues with secondary structure; it was used to design the original tetrahedron reported in [5], and would likely be a good choice for the design of these sequences.

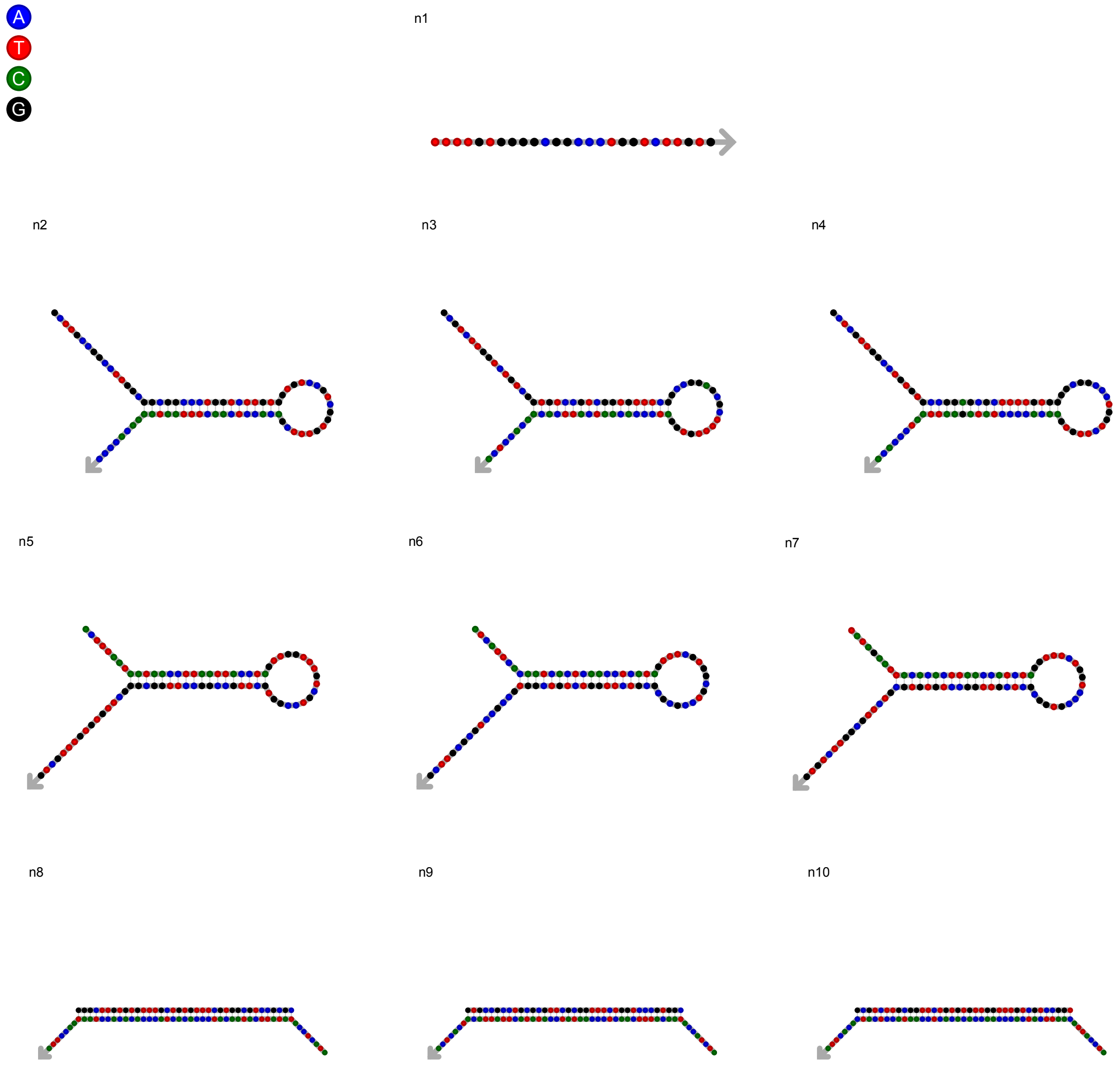

Figure S32: Sequence-tier representation of tetrahedron

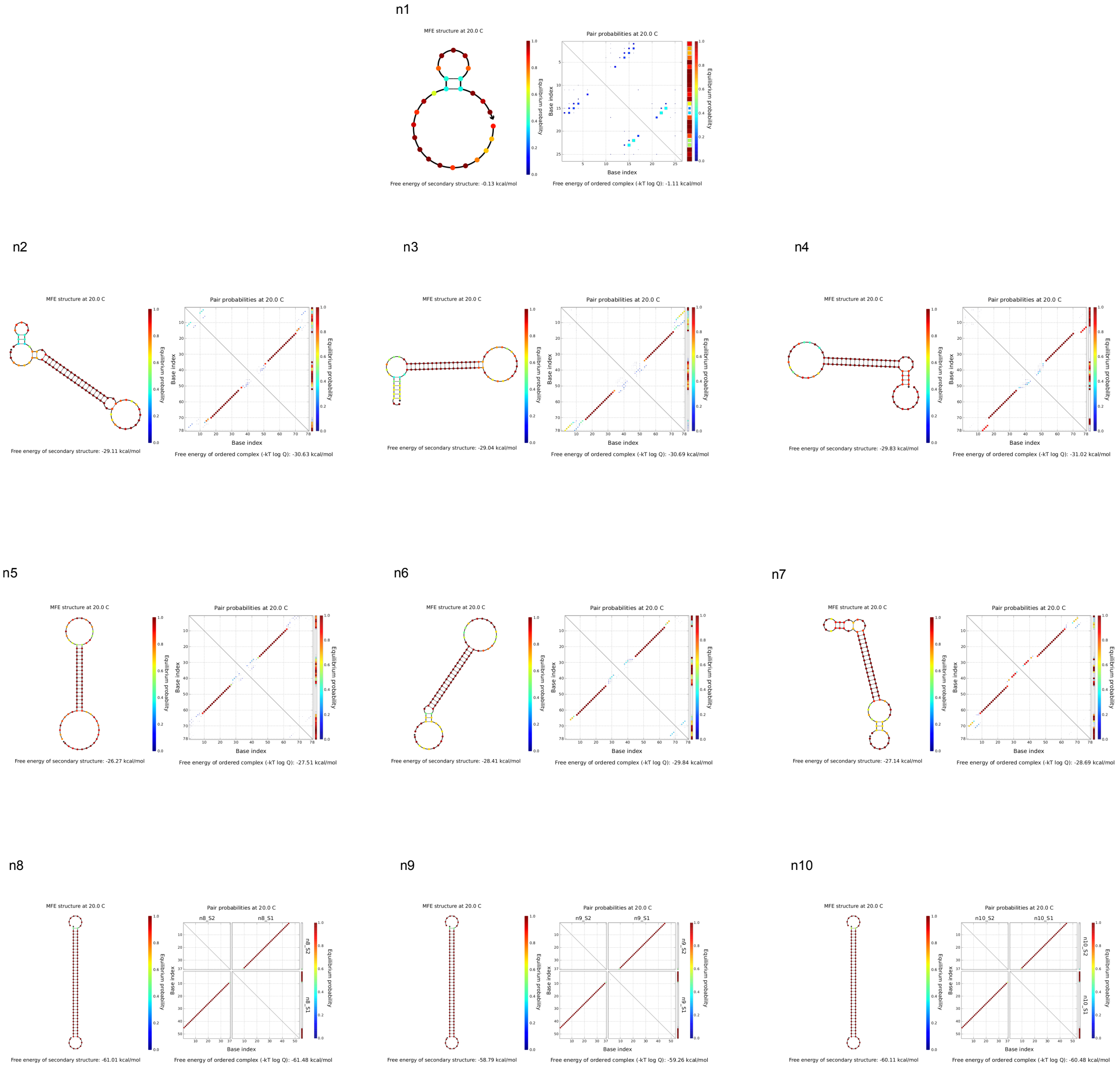

Figure S33: Thermodynamic analysis of DD-designed sequences for the tetrahedron

S2.5 Discussion

In this supplemental section, we have demonstrated the implementation of several important systems using DyNAMiC Workbench. Each of the designed systems has been demonstrated experimentally in the past, making them important and realistic test cases for DyNAMiC Workbench. We verify that DyNAMiC Workbench gives implementations similar to those in previous work, and that the predictions of the reaction enumerator align with experimental evidence. The initial design of these systems was challenging and time-consuming for the authors who first reported them, but design and analysis of all four systems was possible with DyNAMiC Workbench in a matter of hours. For each system, some additional effort would be needed to improve the quality of the designed sequences (possibly with the help of another sequence designer included in DyNAMiC Workbench), but these systems are otherwise ready to be synthesized and tested in a laboratory.

The predictions made by the reaction enumerator align well with experimental evidence, recapitulating the demonstrated behavior of these systems described in the literature. The complexity of the full reaction networks, even for these relatively simple systems, highlights the importance of the reaction condensation algorithm—it would be very difficult to observe even high-level features of the full reaction network. Even the condensed reaction network can be difficult to interpret, and for very large systems (such as those recently demonstrated by Qian and Winfree [39, 40]) one can see how it would become intractable. This motivates the need to develop formal verification methods for automatically checking the full reaction space against the intended behavior specified by the Nodal complementarity graph. Finally, the tetrahedron example demonstrates the limitations of enumerating only unpseudoknotted complexes, and underscores the need to develop methods for handling pseudoknotted intermediates. It may be possible to develop methods for examining a limited subset of pseudoknotted complexes, as has been done for extensions of Turner's thermodynamic model to include pseudoknots [20, 22, 41, 42].

References

[1] Zhang, D. Y., 2011 Towards domain-based sequence design for DNA strand displacement reactions. In *Lect. Notes. Comput. Sc.* (eds. Y. Sakakibara & Y. Mi), pp. 162–175. Springer.

[2] Zadeh, J. N., Steenberg, C. D., Bois, J. S., Wolfe, B. R., Pierce, M. B., Khan, A. R., Dirks, R. M. & Pierce, N. A., 2011 NUPACK: Analysis and design of nucleic acid systems. *J. Comput. Chem.* **32**, 170–173.

[3] Sadowski, J. P. Multisubjective: Better nucleic acid design through fast removal of undesired secondary structure .

[4] Yin, P., Choi, H. M. T., Calvert, C. R. & Pierce, N. A., 2008 Programming biomolecular self-assembly pathways. *Nature* **451**, 318–322.

[5] Sadowski, J. P., Calvert, C. R., Zhang, D. Y., Pierce, N. A. & Yin, P., 2014 Developmental Self-Assembly of a DNA Tetrahedron . *ACS Nano* **8**, 3251–3259.

[6] Zhang, D. Y., 2011 Cooperative hybridization of oligonucleotides. *J. Am. Chem. Soc.* **133**, 1077–1086.

[7] Seelig, G., Soloveichik, D., Zhang, D. Y. & Winfree, E., 2006 Enzyme-Free Nucleic Acid Logic Circuits. *Science* **314**, 1585–1588.

[8] Lakin, M. R., Youssef, S., Cardelli, L. & Phillips, A., 2012 Abstractions for DNA circuit design. *J. R. Soc. Interface* **9**, 470–486.

[9] Wang, Y. L., Mueller, J. E., Kempe, D. & Seeman, N. C., 1991 Assembly and characterization of five-arm and six-arm DNA branched junctions. *Biochemistry* **30**, 5667–5674.

[10] Seeman, N. C., 1982 Nucleic acid junctions and lattices. *J. Theor. Biol.* **99**, 237–247.

[11] Kallenbach, N. R., Ma, R.-I. & Seeman, N. C., 1983 An immobile nucleic acid junction constructed from oligonucleotides. *Nature* **305**, 829–831.

[12] Turberfield, A. J., Mitchell, J. C., Yurke, B., Mills, A. P., Blakey, M. I. & Simmel, F. C., 2003 DNA fuel for free-running nanomachines. *Phys. Rev. Lett.* **90**, 118102.

[13] Seeman, N. C., 2003 DNA in a material world. *Nature* **421**, 427–431.

[14] Chen, J. H. & Seeman, N. C., 1991 Synthesis from DNA of a molecule with the connectivity of a cube. *Nature* **350**, 631–633.

[15] Zhang, Y. & Seeman, N. C., 1994 Construction of a DNA-truncated octahedron. *J. Am. Chem. Soc.* .

[16] Sa-Ardyen, P., Jonoska, N. & Seeman, N. C., 2004 Self-assembly of irregular graphs whose edges are DNA helix axes. *J. Am. Chem. Soc.* **126**, 6648–6657.

[17] Wang, X. & Seeman, N. C., 2007 Assembly and Characterization of 8-Arm and 12-Arm DNA Branched Junctions. *J. Am. Chem. Soc.* **129**, 8169–8176.

[18] Zadeh, J. N., Wolfe, B. R. & Pierce, N. A., 2011 Nucleic acid sequence design via efficient ensemble defect optimization. *J. Comput. Chem.* **32**, 439–452.

[19] Schaeffer, J. M., 2012 *Stochastic simulation of the kinetics of multiple interacting nucleic acid strands*. Ph.D. thesis, Caltech.

[20] Dirks, R. M. & Pierce, N. A., 2003 A partition function algorithm for nucleic acid secondary structure including pseudoknots. *J. Comput. Chem.* **24**, 1664–1677.

[21] Dirks, R. M., Bois, J. S., Schaeffer, J. M., Winfree, E. & Pierce, N. A., 2007 Thermodynamic analysis of interacting nucleic acid strands. *SIAM Rev.* **49**, 65–88.

[22] Dirks, R. M. & Pierce, N. A., 2004 An algorithm for computing nucleic acid base-pairing probabilities including pseudoknots. *J. Comput. Chem.* **25**, 1295–1304.

[23] Zhang, D. Y. & Winfree, E., 2009 Control of DNA strand displacement kinetics using toehold exchange. *J. Am. Chem. Soc.* **131**, 17303–17314.

[24] Dirks, R. M. & Pierce, N. A., 2004 Triggered amplification by hybridization chain reaction. *Proc. Natl. Acad. Sci. U. S. A.* **101**, 15275–15278.

[25] Zhang, D. Y. & Seelig, G., 2011 Dynamic DNA nanotechnology using strand-displacement reactions. *Nat. Chem.* **3**, 103–113.

[26] Zuker, M., 2003 Mfold web server for nucleic acid folding and hybridization prediction. *Nucleic Acids Res.* **31**, 3406–3415.

[27] Gruber, A. R., Lorenz, R., Bernhart, S. H., Neuböck, R. & Hofacker, I. L., 2008 The Vienna RNA websuite. *Nucleic Acids Res.* **36**, W70–4.

[28] Nilsen, T. W., Grayzel, J. & Prensky, W., 1997 Dendritic nucleic acid structures. *J. Theor. Biol.* .

[29] Wang, J., Jiang, M. & Nilsen, T. W., 1998 Dendritic nucleic acid probes for DNA biosensors. *Journal of the American ...* .

[30] Hudson, R. H. E. & Damha, M. J., 1993 Nucleic acid dendrimers: novel biopolymer structures. *J. Am. Chem. Soc.* **115**, 2119–2124.

[31] Horn, T. & Urdea, M. S., 1989 Forks and combs and DNA: the synthesis of branched oligodeoxyribonucleotides. *Nucleic Acids Res.* **17**, 6959–6967.

[32] Li, Y., Tseng, Y. D., Kwon, S. Y., D'Espaux, L., Bunch, J. S., McEuen, P. L. & Luo, D., 2004 Controlled assembly of dendrimer-like DNA. *Nat. Mater.* **3**, 38–42.

[33] Rothemund, P. W. K., 2006 Folding DNA to create nanoscale shapes and patterns. *Nature* **440**, 297–302.

[34] Douglas, S. M., Dietz, H., Liedl, T., Högberg, B., Graf, F. & Shih, W. M., 2009 Self-assembly of DNA into nanoscale three-dimensional shapes. *Nature* **459**, 414–418.

[35] Dietz, H., Douglas, S. M. & Shih, W. M., 2009 Folding DNA into Twisted and Curved Nanoscale Shapes. *Science* **325**, 725–730.

[36] Shih, W. M. & Lin, C., 2010 Knitting complex weaves with DNA origami. *Curr. Opin. Struct. Biol.* **20**, 276–282.

[37] Wei, B., Dai, M. & Yin, P., 2012 Complex shapes self-assembled from single-stranded DNA tiles. *Nature* **485**, 623–626.

[38] Ke, Y., Ong, L. L., Shih, W. M. & Yin, P., 2012 Three-dimensional structures self-assembled from DNA bricks. *Science* **338**, 1177–1183.

[39] Qian, L., Winfree, E. & Bruck, J., 2011 Neural network computation with DNA strand displacement cascades. *Nature* **475**, 368–372.

[40] Qian, L. & Winfree, E., 2011 Scaling up digital circuit computation with DNA strand displacement cascades. *Science* **332**, 1196–1201.

[41] Liu, B., Mathews, D. H. & Turner, D. H., 2010 RNA pseudoknots: folding and finding. *F1000 Biol Rep* **2**, 8.

[42] Möhl, M., Salari, R., Will, S., Backofen, R. & Sahinalp, S. C., 2010 Sparsification of RNA structure prediction including pseudoknots. *Algorithms Mol. Biol.* **5**, 39.
